# MicroRNAs and Glucocorticoid-Induced Apoptosis in Lymphoid Malignancies

**DOI:** 10.1155/2013/348212

**Published:** 2013-01-29

**Authors:** Ronit Vogt Sionov

**Affiliations:** The Department of Biochemistry and Molecular Biology, The Institute for Medical Research-Israel-Canada, Hadassah Medical School, The Hebrew University of Jerusalem, Ein-Kerem, 91120 Jerusalem, Israel

## Abstract

The initial response of lymphoid malignancies to glucocorticoids (GCs) is a critical parameter predicting successful treatment. Although being known as a strong inducer of apoptosis in lymphoid cells for almost a century, the signaling pathways regulating the susceptibility of the cells to GCs are only partly revealed. There is still a need to develop clinical tests that can predict the outcome of GC therapy. In this paper, I discuss important parameters modulating the pro-apoptotic effects of GCs, with a specific emphasis on the microRNA world comprised of small players with big impacts. The journey through the multifaceted complexity of GC-induced apoptosis brings forth explanations for the differential treatment response and raises potential strategies for overcoming drug resistance.

## 1. Introduction

### 1.1. Glucocorticoids in the Treatment of Lymphoid Malignancies

Glucocorticoids (GCs) are among the most effective drugs used in the treatment of hematopoietic malignancies of the lymphoid lineage in virtue of their ability to induce apoptosis of these cancerous cells [1–3]. The main hematopoietic cancer types that respond well to GC therapy include T acute lymphoblastic leukemia (T-ALL), chronic B lymphocytic leukemia (CLL), multiple myeloma (MM), Hodgkin's lymphoma (HL), and non-Hodgkin's lymphoma (NHL). GCs appear, however, to have little value in the treatment of acute or chronic myeloid leukemia (AML/CML). A major drawback of GC therapy is the gradual development of resistance to GC during treatment that limits the clinical utility of this drug. Poor response to a 7-day monotherapy with the GC prednisone is one of the strongest predictors of adverse outcomes in the treatment of pediatric ALL [2, 4]. A great challenge today is to develop strategies that can overcome the drug resistant phenotype. For this purpose it is important to understand the underlying mechanisms of GC resistance and the signaling pathways regulating apoptosis induced by GCs.

Besides inducing apoptosis of lymphoid cells, GCs are used in palliative care. GC treatment produces rapid symptomatic improvements, including relief of fever, sweats, lethargy, weakness, and other nonspecific effects of cancer.GCs decrease the severity of chemotherapy-induced emesis. GCs are also used in the clinics for other medical conditions such as autoimmune diseases, asthma, ulcerative colitis, chronic obstructive pulmonary disease, kidney diseases, and rheumatologic disorders due to their strong anti-inflammatory and immunosuppressive properties. GC therapy is hampered by a variety of metabolic and medical complications, including insulin resistance, diabetes, hypertension, glaucoma, osteoporosis, and osteonecrosis with increased risk of bone fractures [5–10]. Diabetes may develop by direct GC-mediated induction of apoptosis in insulin-producing beta cells of the Langerhans islets [11–13], and osteoporosis may develop due to apoptosis of osteoblasts [14–16]. GCs also suppress cell growth and proliferation processes in the brain [17, 18].

Besides being used as monotherapy at high dosages, GCs are frequently combined with other chemotherapeutic drugs to achieve rapid and more efficient therapeutic effects. For the treatment of T-ALL, GCs such as prednisone, methylprednisolone, and dexamethasone are usually used in combination with other chemotherapeutic drugs such as vincristine, daunorubicine, L-asparaginase, cytosine arabinoside, doxorubicin, and cyclophosphamide. This multidrug regimen prolongs remission, minimizes the long-term use of prednisone, and thus reduces the steroid-mediated adverse effects. 

Typical B-cell chronic lymphocytic leukemia (CLL) in the early stage of progression responds well to combination chemotherapy including an alkylating agent (such as chlorambucil) plus or minus prednisolone.Advanced stages of the disease often require the addition of an anthracycline and a vinca alkaloid for successful therapy. One commonly used combination is cyclophosphamide, doxorubicin, vincristine, and prednisolone, a drug combination termed CHOP. Rituximab, a chimeric monoclonal antibody directed against the B-cell specific antigen CD20, is often added to the therapy, which is here termed R-CHOP. Rituximab is also combined with fludarabine and cyclophosphamide in the treatment of CLL [19, 20]. Another antibody proved to be efficient against CLL in combination with methylprednisolone is alemtuzumab, which targets CD52. This combination is also effective in p53-defective CLLs [21]. However, alemtuzumab was not found to be superior to rituximab [22]. The immunomodulatory drug lenalidomide shows also good activity in relapse/refractory or treatment-naïve CLL [23, 24].

CHOP is also used for non-Hodgkin's lymphomas and anaplastic large cell lymphoma (ALCL). Sometimes interferon-*α*2b is added in the treatment of the former. GCs are also effective for the treatment of Hodgkin's lymphoma. Here, prednisone has been used in combination with carmustine, vincristine (Oncovin), procarbazine (MOPP), and rituximab. Recently, brentuximab vedotin (Adcetris), an antibody directed towards CD30 conjugated with the anti-tubulin chemotherapeutic agent monomethyl auristatin E [25], has been approved for the treatment of Hodgkin's lymphoma and systemic anaplastic large cell lymphoma. CD30 expression is restricted to only a relative small population of activated T and B cells, and therefore this treatment is expected to be more selective for CD30-positive tumor cells. Another monoclonal antibody entered the clinics is epratuzumab, which targets CD22 and is proved to be efficient in the treatment of adult non-Hodgkin's lymphoma as a single agent or in combination with chemotherapy. A phase II clinical trial showed that combining epratuzumab with rituximab and CHOP (ER-CHOP) may have a favorable response on diffusing large B-cell non-Hodgkin lymphoma (DLBCL) [26]. 

Multiple myeloma (MM) has frequently been treated with vincristine, doxorubicine (Adriamycin), and dexamethasone (VAD) or prednisone/melphalan. Bortezomib (Velcade), lenalidomide, and to a lesser extend thalidomide have proven efficient in the treatment of MM in combination with dexamethasone. This is in addition to autologous or allogeneic hematopoietic stem cell transplantation. Lenalidomide is a 4-amino-glutamyl analogue of thalidomide that lacks the neurological side effects of thalidomide and has emerged as a drug with activity against various hematological malignancies [27, 28]. Bortezomib is a selective inhibitor of the 26S proteasome that stabilizes many cell cycle-regulatory proteins. The antitumor effects of bortezomib in lymphoid tumors have been attributed to NF*κ*B inhibition through stabilization of its inhibitor I*κ*B. Other tumors that have been treated with combination chemotherapy involving a GC include medulloblastoma, primitive neuroectodermal tumors, and ependymomas.

### 1.2. Alternative Treatment Approaches for Overcoming GC Resistance

One major obstacle in the therapy of lymphoid malignancies is the appearance of GC resistant cells. Drug resistance may occur at the level of the glucocorticoid receptor (GR) or through alterations in downstream regulatory pathways. In most GC-resistant ALL primary biopsy specimens, GR was found to be functional [29], suggesting that pharmacological intervention may restore drug sensitivity. Several strategies have been developed that aim to overcome drug resistance through specifically targeting anti-apoptotic pathways. Below, three major strategies applicative for GC therapy are discussed.

#### 1.2.1. Targeting Anti-Apoptotic Bcl-2 Members as a Therapeutic Approach for Overcoming GC Resistance

GC resistance may occur due to overexpression of anti-apoptotic proteins of the Bcl-2 superfamily [30, 31]. Among these, Bcl-2, Bcl-X_L_, and Mcl-1 are frequently overexpressed in lymphomas [32]. 

*1.2.1.1. Targeting Bcl-2 with Small Molecular Inhibitors*. Small molecules that target the anti-apoptotic proteins of the Bcl-2 family are attractive drugs that should be able to overcome GC resistance. One example is ABT-737, a BH3 mimetic that inhibits the pro-survival function of Bcl-2, Bcl-X_L_, and Bcl-w and induces apoptosis in a variety of cancer cell types including leukemias [33–35]. Treatment of the lymphoma-prone E*μ*-Myc transgenic mice with ABT-737 prevented the development of Myc-driven lymphomagenesis [36], understating the need for these anti-apoptotic proteins. Combined use of ABT-737 and the dual specificity PI3K/mTOR inhibitor PI-103 led to loss of c-Myc expression and apoptosis of Burkitt's lymphoma cells, whose tumorigenicity is driven by overexpression of the c-Myc gene [37]. 

The pro-apoptotic effect of ABT-737 in CLL depends on sufficient amount of Bcl-2 that tonically sequesters the pro-apoptotic Bim protein [38]. Also, the sensitivity of lymphoma cell lines to Bcl-2 antagonism is directly related to the amount of Bcl-2 primed with Bim [35]. The sequestration of Bim may explain the marked chemosensitivity of CLL and follicular lymphoma (FL) that express abundant Bcl-2 [38]. This drug-responsive condition is termed “primed for death”. 

ABT-737 potentiated the effect of vincristine, dexamethasone, and L-asparaginase (VXL) treatment on ALL cells [39] and could potentiate the effect of the VXL combination in chemoresistant human primary ALL xenografts [40]. This study also shows a synergistic effect between the three components of the VXL regimen. An additive effect was observed in primary MM cells when ABT-737 was combined with dexamethasone [41, 42]. 

ABT-263 (Navitoclax) is a second generation, orally bioavailable small molecule Bcl-2 family protein inhibitor that has entered clinical trials with promising efficacy on CLL [43–46]. ABT-263 has been shown to have synergistic effects with R-CHOP treatment on mantle cell lymphoma [45]. It also synergizes with rapamycin in killing lymphomas [47]. 

*1.2.1.2. Overcoming ABT-737 Resistance by Targeting Mcl-1*. Resistance to ABT-737 occurs in lymphoma cells with high expression of Mcl-1 and/or Bfl-1/A1 [48]. The pro-apoptotic Bim that is displaced from Bcl-2 by ABT-737, becomes captured by either Bfl-1 or Mcl-1. The resistance could be overcome by decreasing the Mcl-1 level with the cyclin-dependent kinase (Cdk) inhibitors flavopiridol and PHA767491 [48], or by inhibiting mTOR complex 1 (mTORC1) [49] or glycolysis [49, 50]. 

Another approach to overcome Mcl-1-dependent resistance is to use the small molecule obatoclax (GX15-070) that has entered clinical trials in the combined treatment of various hematopoietic neoplasms [51–53]. Obatoclax disrupts the interaction between Mcl-1 and its pro-apoptotic counterparts including Bak, Bax, and Noxa [54, 55]. Obatoclax and flavopiridol synergized in overcoming drug resistance in human myeloma cells through a mechanism involving Bim and Noxa [56]. The multikinase inhibitor sorafenib could synergize with Obatoclax in inducing apoptosis in acute myeloid leukemia (AML) through downregulating Mcl-1 [57]. Obatoclax could overcome GC resistance in ALL through induction of apoptosis and autophagy, an effect that depends on the pro-apoptotic Bak and to a certain extent also on Beclin-1 [58, 59], a mammalian orthologue of yeast Atg6 that plays a central role in autophagy [60]. Under certain conditions, cell death induced by Obatoclax and GC may be executed in the absence of both Bax and Bak [59]. Under these conditions, necroptosis ensues necroptosis ensues, a process mediated by RIP-1 (receptor-interacting protein-1) kinase and the cylindromatosis deubiquitinase CYLD [59]. RIP-1 kinase plays a dual role in determining the cell fate. It may promote either cell death or cell survival dependent on its ubiquitinated state, which is regulated by CYLD and A20, two NF*κ*B target genes [61]. Altogether, there is a general consensus that Obatoclax might be a favorable drug that ought to be combined with dexamethasone/prednisone and/or rapamycin to overcome GC resistance in ALL cells and other hematological lymphoid malignancies. 

*1.2.1.3. Overcoming Bcl-2-Mediated Resistance with Small Molecular Inhibitors of XIAP (X-Linked Inhibitor of Apoptosis)*. Bcl-2-mediated resistance in CLL may also be overcome by small molecular inhibitors of the anti-apoptotic XIAP (X-linked inhibitor of apoptosis) when exposed to TRAIL [62, 63]. XIAP and the cellular cIAPs 1 and 2 are expressed at high levels in CLL cells [62, 63]. XIAP inhibitors enhanced Bcl-2 cleavage and induced a conformational change in Bax [62]. Similarly, XIAP inhibitors sensitized ALL for CD95-induced apoptosis [64]. In patients with T-ALL, poor prednisone response was associated with increased XIAP expression [65]. XIAP inhibition using the low-molecular-weight SMAC mimetic LBW242 resulted in increased prednisone-induced apoptosis in vitro [65]. 

#### 1.2.2. Targeting Notch1 as a Therapeutic Approach for Overcoming GC Resistance

Another anti-apoptotic protein that negatively regulates GC-induced apoptosis is Notch1 [66–68]. Notch1 is indispensable for normal T-cell development [69–71] and is an attractive target in the treatment of hematopoietic malignancies of the T lineage [72]. Mice transplanted with bone marrow cells transduced with a constitutively active form of Notch1 develop T-cell neoplasms [73], while mice transgenic for constitutively active form of Notch3 develop thymic lymphomas [74]. Acute lymphoblastic T-cell leukemia is frequently associated with increased Notch signaling [75–79], which may be caused by the chromosomal translocation t(7; 9)(q34; q34.3) [80], gain-of-function mutations of Notch1 [81], and/or mutations in Fbw7 (F-box and WD repeat domain-containing 7), a negative regulator of Notch1 [82]. 

One approach to avoid Notch activation is to prevent its cleavage by the *γ*-secretase complex using *γ*-secretase inhibitors (GSI) [83]. GSIs can induce apoptosis of various lymphoma cell lines [84–87]. However, GSI as a monotherapeutic agent is often insufficient for inducing apoptosis. Rather, GSI can enhance the pro-apoptotic effect of GCs and other chemotherapeutic agents including the mTOR inhibitor rapamycin [84, 88]. GSI restored GR auto-upregulation and induced apoptosis through induction of Bim [88]. GSI does not overcome GC resistance in T-ALL deficient for PTEN [89, 90], supposedly due to elevated Akt activity. The constitutive Akt activation in the absence of PTEN leads to increased glucose metabolism and bypasses the requirement of Notch signaling to sustain cell growth [89]. In this context it should be noted that Notch1 by itself may upregulate the P13K/Akt pathway via its target gene *Hes1* [89]. As PTEN is a target of several microRNAs that are often expressed abnormally in cancer (see Section  2.4.2.3), resistance to GSI may be far more prevalent. GSI is also not efficient in T-ALL carrying activating mutations in Notch1. Nevertheless, GSI compounds, such as PF-03084014, have entered clinical trials for refractory T-ALL [91]. Preclinical data do show a synergistic effect between GSI inhibition and GC in reducing xenografted T-ALL tumor burden [92]. Another concern associated with the clinical use of GSIs is severe toxicity to various organs at therapeutic doses, which may be explained by the broad action of Notch1 as well as *γ*-secretase on various biological systems. The simultaneous use of GCs may prevent the GSI-induced gastrointestinal toxicity via inhibition of goblet cell metaplasia [92]. A more specific inhibition of Notch1 can be achieved by the SAHM1 peptide that prevents Notch-mediated transcription by interfering with the Mastermind-Notch interaction essential for Notch-mediated transcription of target genes [93]. The effect of this peptide on GC sensitivity awaits examination as well as its toxicity. Since Notch signaling is intertwined with the PI3K/Akt/mTOR signaling axis [94–96], the inhibition of the latter has proven to be more efficient in overcoming GC resistance (see Section 1.2.3) and would be a better therapeutic choice.

#### 1.2.3. Targeting Pro-Survival Protein Kinases

Accumulating data show that GC therapy can affect the activity of several protein kinases, and, vice versa, many protein kinases can affect GC-induced apoptosis [30, 31, 97–99]. The mTOR signaling pathway is frequently activated and found to be essential for cell growth and survival in lymphoid malignancies [100–106]. GC resistance frequently appears in malignant cells due to aberrant activation of various protein kinases that exert anti-apoptotic effects [30, 31, 67, 97, 107–109]. One strategy to overcome GC resistance would be to prevent the activities of the PI3K/Akt/mTOR, MEK1/ERK1/2, and other activated protein kinase pathways. The mTOR inhibitor rapamycin especially has proven efficient in sensitizing human GC-resistant T-ALL, B-ALL, MM, and NPM-ALK^+^ (nucleophosmin-anaplastic lymphoma kinase)-DLBCL to GC-induced apoptosis [110–117]. The combinatory therapy of rapamycin with dexamethasone was proven to be effective also in PTEN-negative cells [111]. A lower dose of dexamethasone was sufficient for reducing T-ALL burden in a xenograft model when used together with rapamycin [111]. One major drawback with rapamycin therapy is its immunosuppressive function, which adds to the immunosuppressive function of GCs.

The dual PI3K/mTOR inhibitor NVP-BEZ235 synergistically enhanced cytotoxicity of dexamethasone, doxorubicine, and cytosine arabinoside (AraC), even in GC-resistant ALL cells [118]. NVP-BEZ235 also overcomes bortezomib resistance in mantle cell lymphoma cells [119]. The broad-acting protein kinase staurosporine was especially effective in overcoming GC resistance in mouse lymphomas that overexpressed Notch-1, Bcl-2, and/or Bcl-X_L_ [120]. This sensitization was achieved through prevention of Akt-mediated inhibition of GSK3 [67] and induction of the pro-apoptotic Nur77 [120]. However, staurosporine was less effective on human T-ALL cell lines (unpublished data), which could rather be sensitized to GC by rapamycin. In order to choose the right kinase inhibitor for combinatory therapy, it is important to determine the kinase responsible for GC resistance prior to therapy.

The cyclin-dependent kinase (Cdk) inhibitors flavopiridol (Alvocidib), BMS-387032 (SNS-032), sunitinib, and sorafenib are currently under clinical trials for relapsed/refractory CLL [121]. Multityrosine kinase inhibitors have also been developed for the treatment of lymphoid malignancies. These include Vandetanib (ZD6474), Bosutinib (SKI-606), TKI258 (CHIR-258), Pazopanib (GW786034), and Axitinib (AG013736). CHIR-258, a potent inhibitor of Flt3 (fms-like tyrosine kinase receptor-3), c-Kit tyrosine kinase, and fibroblast growth factor receptor 3 (FGFR3), prevented cell growth of FGFR3-positive human multiple myeloma cell lines and augmented their sensitivity to GC-induced apoptosis [122]. Importantly, neither interleukin-6 (IL-6) nor stromal cells conferred resistance to CHIR-258 [122]. 

Other protein kinase inhibitors with more cell-type specific effects have been developed, which are expected to have less adverse effects. The classical example for efficient use of a specific protein kinase inhibitor in the clinics is the Bcr-Abl kinase inhibitor STI-572 (Imatinib) used for the treatment of chronic myelogenic leukemia (CML) [123]. A similar strong response of a single agent was observed in ALK^+^-anaplastic large cell lymphoma (ALCL) patients treated with Crizotinib, an inhibitor of the ALK tyrosine kinase [124]. Two patients that relapsed after CHOP treatment received Crizotinib as a single agent. Both showed complete response [124].

Another promising target is the B-cell receptor (BCR) signaling, which is important during B-cell oncogenesis and is a key to the survival of malignant B cells, including CLL and DLBCL [125, 126]. The survival of DLBCL may depend on the nonligand-dependent (tonic) signals from the BCR. The BCR signaling can be targeted with small molecular inhibitors directed against Bruton's tyrosine kinase (Btk), spleen tyrosine kinase (Syk), or phosphoinositide 3′-kinase (PI3K) isoform p110*δ* (PI3K*δ*), all being efficient in the treatment of CLL [125]. Targeting Btk with the inhibitor PCI-32765 leads to disruption of BCR signaling and was effective in a preclinical model of B cell non-Hodgin's lymphoma [127, 128]. PCI-32765 seems also to be promising for the treatment of CLL [128–131] and MM [132]. Importantly, PCI-32765 induced apoptosis in CLL cells even in the presence of various exogenous stimuli, including CD40L, BAFF, IL-6, and IL-4 and when cultivated together with stromal cells [131]. Two other Btk inhibitors, Ibrutinib and AVL-263, are also under investigation for CLL [121]. The Syk (spleen tyrosine kinase) inhibitor Fostamatinib had clinical activity in non-Hodgkin lymphoma and CLL [133]. Syk is a cytoplasmic tyrosine kinase that is important for immunoreceptor signaling in B cells. Syk has also been shown to be critical for the survival and maintenance of mature normal and malignant B cells [125, 134] and is frequently expressed at high levels in follicular lymphoma [135]. The PI3K*δ* inhibitor GS-1101 (CAL-101) had preclinical and clinical activity against CLL, mantle cell lymphoma, and MM [121, 129, 136–138]. While the PI3K*α* and *β* isoforms are ubiquitously expressed, PI3K*δ* expression is largely restricted to hematopoietic cells, where it plays a role in B-cell homeostasis and function [139]. PI3Ks are constitutively activated in CLL cells [140–142]. The effect of the Btk, Syk, and PI3K*δ* kinase inhibitors on the sensitivity to GCs warrants investigations. 

Accordi et al. [143] found aberrant activation of protein kinases in poor prognosis pediatric B-cell precursor-ALL patients. The p56^Lck^ (lymphocyte cell-specific tyrosine kinase) activity was enhanced in patients with poor clinical response to prednisone with respect to those with good response [143]. p56^Lck^ is a nonreceptor tyrosine kinase of the Src oncogene family mostly expressed in T cells where it plays an essential role in activation and development, and in some B cells. Its activity is negatively regulated by the membrane-bound tyrosine kinase Csk (c-Src tyrosine kinase). The p56^Lck^ inhibitor Dasatinib (BMS-354825) was shown to enhance apoptosis induction by dexamethasone in otherwise GC-resistant CLL cells [144]. This finding concurs with the observation by Sade et al. [68] showing that Notch-mediated resistance of a mouse lymphoma cell line could be overcome by inhibiting p56^Lck^. In MM, a synergistic effect was observed between the Aurora A kinase inhibitor MNL8237 (Alisertib) and dexamethasone [145]. 

AMPK (AMP activated protein kinase) activation has a dual effect on cell death and survival, which contextually depends on signaling alterations with related oncogenic pathways [146]. MLL-rearranged tumors showed Bcl-2 hyperphosphorylation through AMPK activation [143]. However, in ALL and CLL, activation of AMPK by AICAR (5-Aminoimidazole-4-carboxamide riboside or Acadesine), a cell-permeable nucleotide, induces growth inhibition and apoptosis [146–148]. However, AICAR prevented glucocorticoid-induced apoptosis [149] and thus cannot be combined with steroids in the treatment of lymphoid malignancies. 

Of note, inhibition of either Bcl-2 family members, Notch1, or the Akt/mTOR survival pathways was independently sufficient for sensitizing resistant cells to GC, suggesting a tight crosstalk between these pathways, interruption of one of them being sufficient for abrogating the resistant phenotype. However, it is likely that using a combination of these three strategies together with GC should lead to a more efficient therapy, which may require lower dosages with reduced adverse effects. 

## 2. Parameters Affecting the Susceptibility of Lymphoid Malignancies to GC-Induced Apoptosis

In order to develop strategies to overcome GC resistance, it is essential to understand the signaling network regulating GC-induced apoptosis. Main factors affecting the response to GC include the basal and inducible GR expression levels, the induction of and basal expression of genes involved in the intrinsic apoptotic pathway, the ability of GR to translocate to the mitochondria, the activity of GSK3 (glycogen synthase kinase 3), the general protein kinase activation profile of the cell prior to and following GC therapy, the expression profile of anti-apoptotic proteins, and the activities of pro-survival signaling pathways. The main traits will only be briefly described here as these have been extensively reviewed elsewhere [30, 31, 99, 150–153], and the scope of this paper is to provide updated data with a specific focus on the microRNA world that has emerged to comprise important regulators of most biological processes.

### 2.1. Sufficient Expression Levels of the Glucocorticoid Receptor (GR/NR3C1)

Numerous factors have been shown to affect GC responsiveness by regulating glucocorticoid receptor (GR) activity and expression level. These include GR co-activators and corepressors [154, 155], GR splice variants [156–159], GR isoforms [160, 161], and regulators of GC nucleocytoplasmic shuttle [162–164]. 

The transcription of human GR is regulated by at least 11 different promoters (1A1, 1A2, 1A3, 1B, 1C, 1D, 1E, 1F, 1H, 1I, and 1J) [155, 165], seven of them being embedded in a highly enriched CpG island region subjected to methylation and harbor single nucleotide polymorphisms (SNPs) that affect their activity [166]. Promoter 1A is involved in the upregulation of GR by GC in some kinds of T cells, while downregulated in other cell types [167–169]. GC resistance in primary pediatric T- and B-ALL could not be correlated with either basal or stimulated expression of the 1A-, 1B, or 1C transcripts [170]. 

The GR expression level prior and following GC therapy affects drug responsiveness. The cellular response to GCs depends on sufficient GR expression [30, 171–179], and resistance to GC therapy has been associated with downregulation and loss of GR expression in malignant plasma cells [180, 181]. However, most primary ALL cells showed upregulation of GR expression upon prednisolone treatment regardless of their phenotype or sensitivity to GC-induced apoptosis, suggesting that other factors are more dominant for conferring a GC-resistant phenotype in these cells [29, 170, 182–184]. Many glucocorticoid-regulated genes (e.g., FKBP5 and SOCS1) were upregulated by dexamethasone in all primary ALL xenografts tested, suggesting for a functional GR in these leukemic cells [29]. Also, Beesley et al. [185] observed that receptor mutation is not a common mechanism of GC resistant in primary ALL [185]. However, the minor C allele of rs10482605 (1C) has been associated with a higher complication rate in childhood ALL [186]. A BclI polymorphism in the NR3C1 gene was associated with increased lymphocyte response to methylprednisolone [187]. Also, initial good responder cells may develop resistance upon repeated GC dosages, a phenomenon that sometimes occurs due to downregulation of GR [156, 179, 188, 189]. Regulation of GR expression by microRNAs is discussed in Section 4.1.

Posttranslational modifications of GR are another way of regulating its target gene specificity and involve several cell-signaling cascades [30, 190, 191]. GR can be phosphorylated at Ser211 by CDKs and p38 MAP kinase, and at Ser226 by JNK. Phosphorylation of GR modulates its transcriptional activity, alters its protein stability and subcellular location [192–195]. GR phosphorylation appears to be cell-cycle dependent [196, 197] and may affect GC-sensitivity of T-ALL cells [98, 195].

### 2.2. The Ability to Upregulate the Pro-Apoptotic Gene Bim in Response to GC

#### 2.2.1. GR as a Transcription Factor

GR is a well-known regulator of transcription. In the absence of ligand, GR is mostly located to the cytosol sequestered to heat-shock protein complexes [30, 162]. Following GC binding to GR, the receptor undergoes phosphorylation, dissociates from the heat-shock complexes, dimerizes, and translocates to the nucleus where it either promotes or represses a whole series of genes. Transcriptional activation is either directly mediated by binding of GR to glucocorticoid response elements (GREs), or through interaction with other transcription factors such as forkhead transcription factors, thereby increasing their transcriptional activity on target genes. GR may repress gene expression either through binding to negative GREs (nGREs) or through interaction with and inhibition of the transcription factors activating protein-1 (AP-1) and NF*κ*B. The O-GlcNAc transferase (OGT) was found to be involved in GC-mediated transrepression [198]. Hundreds of genes are regulated by GCs [199–203], and some genes are differentially regulated in GC-sensitive versus GC-resistant cells [29, 199, 204]. 

#### 2.2.2. Importance of Bim in GC-Induced Apoptosis

Of special importance is the induction of the pro-apoptotic Bim (BH3-only B-cell lymphoma 2 (Bcl-2) interacting mediator of cell death; or BCL2L11—Bcl-2-like apoptosis initiator-11) for achieving the propensity to undergo apoptosis in response to GC [29, 30, 67, 205–208]. The central role of Bim in GC-induced apoptosis is understated by the partial GC response of Bim^−/−^ thymocytes [205], and GC resistance of lymphoma cells after knocking down Bim [67, 207]. Bim is often expressed at high basal levels in lymphoid cells [30, 120, 209, 210], and in these cells there is no further need for upregulating Bim in order to achieve an apoptotic response to GCs [30, 59]. However, in several T-ALL and B-ALL cells, an upregulation of Bim in response to GCs is an absolute must, especially when the basal level is low.

Bim was shown to be upregulated in GC-sensitive primary T-ALL samples, but not in resistant ones [29, 182]. Also, a comparison of established T-ALL cell lines, Bim was upregulated in the sensitive ones only [211]. When sufficient Bim expression cannot be achieved, GC resistance pursued. A significantly lower Bim expression was detected in high risk childhood ALL patients who exhibited slow early response to a standard 4-drug induction regimen compared with patients who responded rapidly [212].

Homozygous deletion of Bim has been seen in many mantle cell lymphomas [213] and silencing of Bim by promoter methylation and mutation is common in B-cell lymphomas [214]. However, in pediatric ALL, no correlation between Bim CpG methylation and GC resistance was found [29]. Rather, GC resistance in primary pediatric ALL samples correlated with decreased histone H3 acetylation [29]. The histone deacetylase inhibitor vorinostat relieved Bim repression and exerted synergistic antileukemic efficacy with dexamethasone both in vitro and in vivo using a xenograft model [29]. Bim has been shown to be a prognostic biomarker for early prednisolone response in pediatric ALL [4].

#### 2.2.3. The Pro-Apoptotic Function of Bim and Other Proteins in GC-Induced Apoptosis

Bim is a potent pro-apoptotic protein belonging to the Bcl-2 protein family [215, 216]. Bim binds to the pro-survival proteins Bcl-2, Bcl-X_L_, and Mcl-1, thereby allowing Bax and Bak to promote apoptosis [217]. Bim may also directly bind to Bax and Bak, triggering a conformational change required for their subsequent oligomerization on the mitochondrial outer membrane [215]. Bim appears in various alternative splice variants, which exhibit different intrinsic toxicities and modes of regulation [218]. In GC-resistant primary CLL, Bim was upregulated by dexamethasone, but failed to activate Bax and Bak due to exclusive sequestration to Bcl-2 [219].

Bim may cooperate with the pro-apoptotic PUMA (p53 upregulated modulator of apoptosis) in mediating apoptosis induced by dexamethasone [220]. In B-lymphoid cells, Bmf (Bcl-2 modifying factor) is also important for GC-induced apoptosis [221]. Other pro-apoptotic members of the Bcl-2 family that is not directly upregulated by GCs, but may contribute to the cell death response, include Bid, Bad, and Noxa. Essential downstream mediators are Bak and Bax [222] that are activated by Bim. Also the thioredoxin-interacting protein Txnip (VDUP1/TBP-2) has been shown to be upregulated by GC and could contribute to GC-induced apoptosis in one mouse lymphoma cell line [223]. During GC monotherapy of childhood ALL, GC was found to repress the expression of the pro-apoptotic PMAIP/Noxa, which could be one mechanism leading to impaired GC sensitivity [224]. Conditional overexpression of Noxa restored GC sensitivity [224]. Another transcript of the Bim locus, termed “Bam,” is also induced by GCs in ALL cells, but its importance in GC-induced apoptosis is still not defined [225].

#### 2.2.4. Regulation of Bim Expression by Transcription Factors

**Figure 1:**
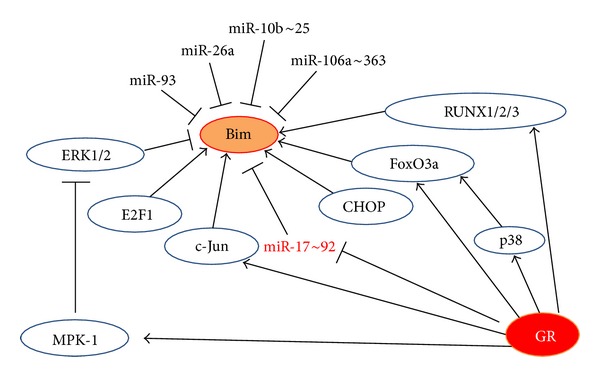
Regulation of Bim expression. Details are described in Sections 2.2.3–2.2.5.

Bim expression is tightly regulated both at the transcription and posttranscriptional levels [215, 218] (Figure 1). No GRE element has been found in the Bim promoter. Rather, GC-induced Bim expression in lymphoid cells requires p38 activation and is mediated by the forkhead transcription factor FoxO3a/FKHR-L1 [226]. FoxO3a has also been shown to promote Bim transcription in various other cellular systems [227–229] and may cooperate with Runx1 (Runt-related transcription factor 1) [230]. Differential recruitment of FoxO3a to the Bim promoter was observed after dexamethasone treatment of GC-sensitive versus GC-resistant childhood ALL xenografts [29]. FoxO3 was found to be an immediate early GR target, whose transcription is further enhanced by stimuli that activate the AMP-activated protein kinase AMPK [231]. The activity of FoxO transcription factors is tightly regulated, inhibited by Akt and ERK signaling, while promoted by p38 signaling [232–236].

Both ERK1/2 and Akt antagonize apoptosis by reducing the Bim expression level. ERK1/2 also directly phosphorylates Bim leading to its proteosomal-dependent degradation [237]. The ribosomal protein S6 kinase (RSK) activated downstream of ERK1/2, phosphorylates BimEL, providing a binding site for the F-box proteins beta-transducin repeat containing protein (*β*TrCP)1 and *β*TrCP2, which promote the polyubiquitination of BimEL [238]. ERK1/2 phosphorylates BimEL at Ser55, Ser69, and Ser73. The ERK1/2-mediated phosphorylation of BimEL at Ser69 facilitates optimal phosphorylation by RSK at Ser93, Ser94, and Ser98 and this motif serves as the binding sites for *β*TrCP1/2 [238]. While ERK1/2 lowers the affinity of Bim for Mcl-1 and Bcl-X_L_ and targets Bim for degradation [239], phosphorylation of Bim by JNK increases the pro-apoptotic activity of Bim [240, 241]. GCs may repress ERK1/2 activity through upregulation of mitogen-activated protein kinase phosphatase 1 (MKP-1) [242]. Several drugs that inhibit the ERK1/2 and PKB/Akt pathways may facilitate upregulation of Bim expression. MEK inhibitor-induced Bim expression per se is usually insufficient to promote apoptosis. Additional signals are required, such as simultaneous inhibition of the PKB/Akt pathway or the downstream mammalian target of rapamycin (mTOR) kinase [218]. Apoptosis may be induced in a variety of ALL cells when cotreated with dexamethasone and a MEK/ERK inhibitor or an Akt inhibitor [67, 108, 243].

Early studies by the Thompson research group noticed that c-Jun played a role in GC-induced apoptosis [244]. An increase in c-Jun was observed in GC-sensitive, but not GC-resistant T-ALL cell lines, while c-Fos and JunD were unaffected by the steroid. Antisense to c-Jun conferred GC resistance [244]. Recently, the c-Jun issue was revisited. Chen et al. [204] reconfirmed that c-Jun was upregulated by GCs in GC-sensitive, but not GC-resistant ALL cells. They further showed that c-Jun is recruited to the AP-1 site of the Bim promoter upon GC treatment [204]. Another study showed that dexamethasone-induced Bim expression was decreased in cells harboring a dominant-negative c-Jun [245], suggesting a role for c-Jun in the upregulation of Bim. This research group also found a Runx2-dependent upregulation of Bim. A p38 inhibitor prevented dexamethasone-induced expression of Runx2, c-Jun, and Bim, suggesting that p38-MAPK activation acts upstream to the induction of these three molecules [245].

#### 2.2.5. Regulation of Bim Expression by MicroRNAs

Another level of Bim regulation is through microRNAs. Bim transcription is repressed by the miR-17~92 microRNA cluster [246], which, in turn, is repressed by GCs [206]. Thus, one mechanism by which GCs upregulate Bim is through repression of miR-17~92. Of note, the miR-17~92 cluster is often overexpressed or amplified in human cancers [247–252], thereby preventing the upregulation of Bim required for an apoptotic response. Another microRNA that suppresses Bim expression is miR-26a, which is frequently upregulated in T-ALL patients [253]. In gastric cancer, miR-106a~363 targets Bim [254]. The miR-106a~363 cluster located at chromosome Xq26.2 is the paralogue of miR-17~92 and encodes for miR-363, miR-106a, and miR-20b [255]. In hepatocellular carcinoma, miR-25 of the miR-106b~25 cluster targets Bim [256]. Also, the miR-106b~25 cluster, which includes miR-106b, miR-93 and miR-25, is a paralogue of the miR-17~92 cluster and located on chromosome 7 within the thirteenth intron of the protein-coding gene Mcm7.

#### 2.2.6. Regulation of FoxO Transcription Factors by MicroRNAs

**Figure 2:**
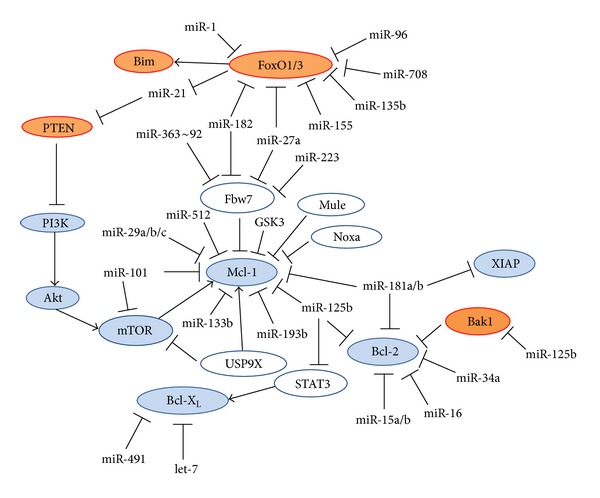
Interplay between microRNAs, pro- and anti-apoptotic proteins affecting GC-induced apoptosis. Details are described in Sections 2.2.6, 2.4.2, and 2.5.

Also, the FoxO transcription factors, important for Bim upregulation, are regulated by microRNAs [257] (Figure 2). FoxO1 and FoxO3 transcripts might be targeted by miR-182 [258–261], miR-1 [262], miR-27a [258], miR-96 [258], and miR-155 [263, 264]. miR-155 plays a role in the activation and function of B and T lymphocytes [265, 266] (see Section 3.1.6). miR-182 is upregulated in several human lymphoid cell lines [261]. miR-182 expression was higher in GC-resistant cells in comparison to GC sensitive ones [261]. Increased expression of miR-182 reduced total FoxO3a expression in T-ALL cells with consequent lower Bim expression. FoxO3a and Bim increased upon downregulation of miR-182, suggesting that miR-182 is involved in conferring GC resistance [261].

The expression of the miR-182~96~183 cluster was induced in splenocytes from mouse with experimental systemic lupus erythematosus (SLE) [267], suggesting a role of these microRNAs in the breakdown of immunological tolerance and the manifestation of chronic autoimmune inflammation. This microRNA cluster was also upregulated upon T-cell activation by an IL-2-dependent manner. Prevention of the expression of the miR-182~96~183 cluster led to increased FoxO1 expression and limited population expansion of activated T-helper cells, due to increased cell death [260].

Vice versa, FoxO3a was found to negatively regulate the oncomiR miR-21, which may be one mechanism by which FoxO3a regulates apoptosis [268]. As miR-21 targets PTEN [269, 270], activation of FoxO3 by GCs [271] may be one mechanism responsible for the GC-induced reduction in Akt activity.

### 2.3. Mitochondrial Translocation of GR

Besides function as a transcription factor in the nucleus, GR was found to translocate to the mitochondria in GC-sensitive, but not GC-resistant, lymphoma cell lines [272]. GR was also found to translocate to the mitochondria in GC-sensitive thymocytes [272, 273]. Although there is one paper describing an interaction between GR and Bcl-2 in the mitochondria [274], GC-induced mitochondrial GR translocation in GC-sensitive thymocytes and lymphoma cells proceeded in the absence of Bcl-2 [272]. Exclusive overexpression of GR in the mitochondria was sufficient for inducing apoptosis [272], suggesting that mitochondrial GR may contribute to GC-induced apoptosis.

Glucocorticoids are known to exert multiple effects on the mitochondria. Glucocorticoid treatment inhibited Complex I and Complex III of the electron transport chain, and the mitochondria was found to be the primary source of H_2_O_2_ production required for GC-induced apoptosis of lymphoma cells [275, 276]. GCs may interact with the mitochondrial thioredoxin Trx2, a redox regulator [277], and directly modulate mitochondrial gene transcription [278]. Several mitochondrial metabolite and protein transporters and two subunits of the ATP synthase were downregulated in T-ALL and precursor B-ALL cells at the gene expression level by dexamethasone. These changes were observed in GC-sensitive, but not GC-resistant, cells [279]. Corticosterone and other steroids were found to directly act on mitochondria to inhibit mitochondrial ATP production by suppressing electron transfer from NADH to the electron transfer chain through complex I [280].

### 2.4. The Kinome

The cellular protein kinase network (kinome) has critical influence on the GC sensitivity of lymphoid cells [30, 31, 97, 281]. Above, I discussed the importance of p38 in Bim induction and activity. Below, I will provide data supporting an involvement of GSK3 (glycogen synthase kinase 3) in GC-induced apoptosis, and the antagonism of its activity by protein kinases such as Akt and mTOR, which leads to GC resistance. 

#### 2.4.1. GSK3 (Glycogen Synthase Kinase 3) Activity

The activity of GSK3 was found to be essential for GC-induced apoptosis [67, 282]. GSK3 inhibitors prevented GC-induced apoptosis, and GC resistance frequently occurs through inhibition of GSK activity. Reactivating GSK3 by using inhibitors of the PI3K-Akt or mTOR pathways sensitized GC-resistant cells to GC-induced apoptosis [67, 108, 115, 116, 243, 283]. GSK3*α* was found to interact with GR in the absence of ligand and released from GR following exposure to GC [67]. GC treatment led to interaction of both GSK3*α* and GSK3*β* with Bim [67]. GSK3*β* also regulates GR transcriptional activity of Bim, IAP1 (Inhibitor of Apoptosis 1), and GILZ (glucocorticoid-induced leucine zipper) [282, 284]. This effect of GSK3 on GR transactivation was independent of known GSK3*β* phosphorylation sites [284]. GSK3*β* was also shown to be involved in GC-induced bone lost [285].

#### 2.4.2. Activity of the PI3K-PKB/Akt, mTOR, and ERK Pro-Survival Pathways

The PI3K/Akt and mTOR signaling pathways are frequently hyperactivated in GC-resistant T-ALL [104, 286, 287] and is associated with poor prognosis and chemotherapeutic resistance in pediatric B-precursor ALL [288]. mTOR is a crucial regulator of cell metabolism, growth, and proliferation and mTOR is positively regulated by PI3K/Akt and Notch1 [96, 289], while negatively regulated by the tuberous sclerosis tumor suppressor complex (TSC1/TSC2). mTORC2 activity was essential for Notch-driven T lymphomagenesis [290]. Activation of mTOR contributes to tumor cell survival in ALK (anaplastic lymphoma kinase)-positive ALCL (anaplastic large cell lymphoma) [102], mantle cell lymphoma [103], childhood B-precursor ALL [112], T-ALL [110], and AML [291]. Akt and mTOR confer drug resistance by phosphorylating a series of targets [292, 293]. Phosphorylation and inactivation of GSK3 is a major cause for GC resistance [67] that can be overcome by reactivating GSK3, for example, by Akt inhibitors or mTOR inhibitors. As mentioned in Section 1.2.3, the mTOR inhibitor Rapamycin is efficient in overcoming GC resistance in various lymphoid malignancies. GC resistance can also be overcome in Akt-active lymphoma cells by inhibiting Src members (e.g., by PP1), PI3K (e.g., Wortmannin), or an Akt inhibitor [67, 68].

Combination of GC with rapamycin or GC with Obatoclax led to reduced Akt phosphorylation at Ser473 [59], suggesting that mTOR may also act upstream to Akt [294]. mTORC1 directly phosphorylates Akt/PKB on Ser473 and facilitates Thr308 phosphorylation by PDK1 [295]. GCs could also independent of other cytotoxic agents reduce mTOR activity in lymphoid cells [296]. Low-dose arsenic trioxide could sensitize GC-resistant ALL to Dex through an Akt-dependent pathway [286]. Inhibition of mTOR with rapamycin, which binds to FKBP12, leads to increased Bim expression and overcomes Ras-dependent survival signals [297]. Synergy between mTOR inhibitors (e.g., rapamycin (Sirolimus) and CCI-779 (Temsirolimus)) and other chemotherapeutic agents has been observed in B- and T-lineage ALL cell lines and preclinical models [96, 298]. 

*2.4.2.1. Negative Regulation of Akt by PTEN*. The Akt activity is negatively regulated by PTEN (phosphatase and tensin homolog deleted on chromosome 10), a tumor suppressor gene that is suppressed, mutated, or deleted at high frequency in a large number of cancers [299]. PTEN mutations or deletions are frequent in T-ALL and PTEN deletions are associated with less favorable outcome in T-ALL [104, 300]. The PTEN status of the cell affects drug sensitivity. For instance, treatment of T-ALL with gamma secretase inhibitor (GSI) was only efficient if the cells expressed functional PTEN [90]. One mechanism by which Notch confers GC resistance is through PTEN inhibition leading to Akt activation. PTEN specifically catalyzes the dephosphorylation of 3′-phosphate of the inositol ring in phosphatidylinositol (3,4,5)-triphosphate (PIP_3_) resulting in the biphosphate product phosphatidyl (4,5)-biphosphate (PIP_2_). PIP_3_ is a second messenger generated by PI3K that binds to the pleckstrin homology (PH) domain of Akt, which allows its phosphorylation and activation by the 3-phosphoinositide-dependent protein kinase 1 (PDK1) [292]. 

*2.4.2.2. Regulation of PTEN Stability by Phosphorylation and Ubiquitination*. Taken into account the important role of PTEN in determining drug sensitivity, mechanisms regulating PTEN activity and stability have strong impact on the drug response. PTEN is regulated by several mechanisms [301]. Besides gene mutation and deletion, reduced PTEN expression has been attributed to epigenetic events such as promoter methylation [302, 303]. At the posttranslational level, phosphorylation and ubiquitination decrease PTEN protein levels, while oxidation and acetylation reduce PTEN activity [301]. Rak phosphorylation of PTEN at Tyr336 stabilizes the PTEN protein [304], while phosphorylation at Thr366, Ser370, Ser380, Thr382, and Ser385 by casein kinase 2 (CK2) and GSK3*β* reduces its stability [305, 306].

PTEN is regulated by the protooncogene ubiquitin ligase NEDD4-1 (neural precursor cell expressed, developmentally downregulated 4) that promotes PTEN for proteasomal degradation [307]. In multiple human cancer samples where the genetic background of PTEN was normal, but its protein level was low, NEDD4-1 was highly expressed [307]. Upon TCR/CD28 stimulation of T cells, PTEN undergoes inactivation by NEDD4-1 [308]. The association between PTEN and NEDD4 could be impeded by the E3 ubiquitin ligase Cbl-b (Casitas-B-lineage lymphoma protein-b) [308]. Cbl-b^−/−^ T cells show elevated Akt activity, which was abrogated by simultaneous deficiency in NEDD4 [308]. PTEN is also negatively regulated by the anti-apoptotic XIAP (X-linked inhibitor of apoptosis) that promotes PTEN for polyubiquitination and proteosomal degradation [309]. Induction of apoptosis in B-CLL by arsenic trioxide was shown to lead to activation of c-Jun-NH_2_ terminal kinase (JNK), inactivation of AKT and NF*κ*B, XIAP downregulation, and PTEN upregulation [310]. Two other E3 ligases downregulating PTEN include WWP2 (WW-domain containing protein-2 or AIP-2, atrophin-1-interacting protein 2) [311], and CHIP (chaperone-associated E3 ligase C terminus of Hsc70-interacting protein) [312]. Recently, PTEN was shown to be upregulated by dexamethasone [313]. 

**Figure 3:**
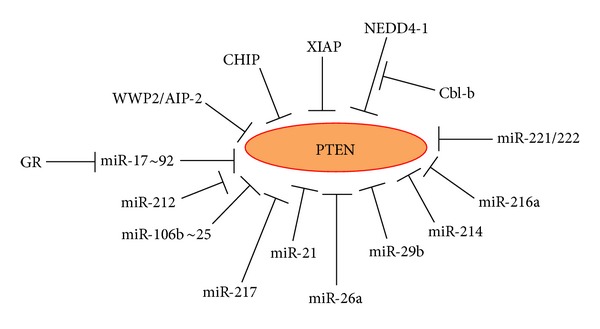
Regulation of PTEN expression. Details are described in Sections  2.4.2.1–2.4.2.3.

*2.4.2.3. Regulation of PTEN Stability by MicroRNAs*. PTEN expression can also be repressed by a range of microRNAs including the miR-17~92 cluster [247, 248], miR-106b~25 [314], miR-21 [269], miR-26a [253, 315], miR-29b [316], miR-214 [317, 318], miR-216a and miR-217 [319], miR-212 [320], miR-221, and miR-222 [321] (Figure 3).

### 2.5. Expression Levels of Anti-Apoptotic Proteins of the Bcl-2 Superfamily

#### 2.5.1. Bcl-2 and Bcl-*X*_*L*_

Bcl-2 and Bcl-X_L_ are anti-apoptotic proteins residing in the mitochondrial outer membrane and in the endoplasmic reticulum. They prevent apoptosis of various chemotherapeutic drugs including GCs by capturing pro-apoptotic members of the Bcl-2 superfamily, including Bim, Bax, and Bak [215, 322, 323]. Bcl-2 may also regulate gene expression [324, 325], cell cycle [326–328], activate ERK1/2 [324, 329], and modulate the activities of transcription factors such as p53 [330], E2F [325], NF*κ*B [331], and Notch [332, 333]. Bcl-2 promotes T-cell lymphoma in a p27^Kip1^-deficient background [334]. This may be explained by the ability of Bcl-2 to modulate p27^Kip1^ expression and promote G_0_ arrest [325, 327, 331, 335, 336].

Long-term exposure to GCs could overcome resistance caused by either Bcl-2 or Bcl-X_L_ [30, 120, 337]. Overexpression of Bcl-2 is common in leukemias and lymphomas [338–341]. In follicular lymphoma (FL) and diffuse large B-cell lymphoma (DLBCL), Bcl-2 upregulation is commonly due to the t(14,18)(q32; q21) translocation, which places the Bcl-2 gene under the control of Ig heavy chain enhancers [342–344]. 

*2.5.1.1. Targeting of Bcl-2 by MicroRNAs*. Overexpression of Bcl-2 is common in CLL due to the loss or downregulation of the human chromosome 13q14 locus, which harbors the miR-15a and miR-16-1 cluster [345]. These microRNAs directly target the anti-apoptotic Bcl-2 protein [346]. Overexpression of either microRNA was sufficient to completely abrogate Bcl-2 expression in CLL cells. Overexpression of miR-15a and miR-16-1 in CLL cells led to cleavage of procaspase-9 and PARP (poly-ADP-ribose polymerase) and activation of the intrinsic apoptosis pathway. These two microRNAs could serve as natural antisense Bcl-2 actors that have potential use in the therapy of Bcl-2 overexpressing tumors [346].

The tumor-suppressor miR-34a, a pivotal member of the p53 network, also downregulates Bcl-2 [347, 348], which may be one mechanism by which p53 activation leads to downregulation of Bcl-2. Recent studies suggest that miR-125b also may contribute to Bcl-2 repression [349–351]. It also targets Mcl-1 and Bcl-w, and indirectly Bcl-X_L_ by attenuating IL-6/STAT-3 (signal transducer and activator of transcription 3) signaling pathway [350, 352]. miR-125b may function both as tumor suppressor and as an oncogene [350] and has been widely considered as conferring drug resistance, among others by downregulating Bak1 (Bcl-2 antagonist killer 1) [353–355] and Bmf [356]. Over-expression of miR-125b could induce leukemia in a mouse model [357].

miR-181a/b that shows altered expression in CLL could also target Bcl-2, besides acting on Mcl-1 and XIAP [358–360]. Bcl-X_L_ can be targeted by the tumor suppressor microRNA let-7 [361] and miR-491 [362]. A putative GR binding site was found within the promoter region of let7a2 [363].

#### 2.5.2. Mcl-1

A predominant feature of the gene expression signature leading to GC resistance in ALL was found to be elevated expression of the anti-apoptotic Mcl-1 (myeloid cell leukemia sequence 1) [364, 365]. Mcl-1 expression is especially high in MLL-rearranged ALL, which represents an unfavorable type of leukemia that is often highly resistant to GCs [365]. Mcl-1 is also frequently overexpressed in B-cell and mantle-cell lymphomas, CML, CLL, and MM. Mcl-1 expression renders cancer cells resistant to the Bcl-2 antagonist ABT-737.

Mcl-1 is an anti-apoptotic protein that sequesters the pro-apoptotic proteins tBid, Bim, Puma, Noxa, and Bak [366]. Besides preventing GC-induced apoptosis [287], Mcl-1 confers resistance to TRAIL (tumor necrosis factor-related apoptosis inducing ligand)-induced cell death [367]. 

*2.5.2.1. Regulation of Mcl-1 Stability*. Mcl-1 differs from Bcl-2 and Bcl-X_L_ in having a short protein turnover regulated by the 26S proteasome and its expression is tightly regulated [368]. Unlike Bcl-2, chromosomal translocations have not been implicated in dysregulated Mcl-1 levels. Rather, cellular signaling regulates Mcl-1 function and expression at the posttranslational level.

Rapamycin, a mTOR inhibitor that sensitizes resistant ALL cells to GC, reduces the expression level of Mcl-1 [113, 287]. Mcl-1 level could also be reduced by the protein kinase inhibitor Sorafenib. The degradation of Mcl-1 depends on GSK3-mediated phosphorylation of Mcl-1 at Ser159 [369, 370]. E3 ubiquitin ligases implicated in the regulation of Mcl-1 include Mule (Mcl-1-ubiquitinase ligase E3) [371], SCF^*β*-TrCP^ (Skp1/Cul1/F-box protein *β*-transducin repeat-containing protein) [369], and Fbw7 (F-box and WD repeat domain-containing 7) which is part of the Skp1-Cullin1-F-box (SCF) E3 ligase complex [372]. The deubiquitinase USP9X (ubiquitin specific peptidase 9 X-linked) is an important regulator of Mcl-1 stability [373]. Silencing of USP9X resulted in loss of Mcl-1. USP9X removes degradative Lys48-linked polyubiquitin chains on Mcl-1. High levels of Mcl-1 correlated with elevated USP9X expression in follicular lymphoma, diffuse large B-cell lymphoma, and some other cancer samples. Increased expression of USP9X mRNA was associated with poor prognosis of multiple myeloma [373]. USP9X also interacts with mTOR, negatively regulating its activity [374].

Interaction with BH3-only family members may also affect Mcl-1 stability. Whereas Noxa may destabilize Mcl-1, Bim increases its stabilization [375]. Noxa-induced degradation of Mcl-1 requires the E3 ligase Mule. Overexpression of Noxa triggered an increase in the Mule/Mcl-1 interaction in parallel to a decrease in Mule/USP9X complex formation [376].

In an Akt-driven, E*μ*-Myc lymphoma mouse model, translational regulation of Mcl-1 by mTOR has been implicated in promoting lymphomagenesis [377]. As GC may activate GSK3 [67] and GSK3 inhibits mTOR through phosphorylation of TSC2 [378] and promotes Mcl-1 degradation [369, 370], Mcl-1 expressing lymphoid cells may ultimately undergo apoptosis if the exposure time to GC is sufficiently long. This may explain why many Mcl-1-positive ALL cells exhibit delayed response to GCs, and not complete resistance [67, 108]. Also, the anti-apoptotic function of Mcl-1 appears to require simultaneous expression of other anti-apoptotic Bcl-2 family members [379]. Similarly, overexpression of Mcl-1 in Bcl-2- and Bcl-X_L_-negative mouse double positive thymic lymphoma cells did not confer GC resistance upon these cells [120]. Usually, Mcl-1 is expressed together with other anti-apoptotic proteins in GC-resistant lymphoid malignancies.

*2.5.2.2. Regulation of Mcl-1 by MicroRNAs*. Mcl-1 is also regulated by microRNAs (Figure 2), including miR-29a [380], miR-29b [381–383], miR-101 [384], miR-125b [350], miR-181a/b [358, 385], miR-133b [386], miR-193b [387], and miR-512 [388]. ALK-positive anaplastic large cell lymphomas (ALCL) express low levels of miR-29a, whose downregulation requires an active NPM-ALK kinase, and may probably also be due to methylation repression [380]. Enforced miR-29a expression reduced Mcl-1 expression in ALCL cells and reduced tumor growth in a xenografted model [380]. miR-29b is downregulated in primary MM and AML samples and forced overexpression of miR-29b-induced apoptosis in MM and AML cells [381, 383]. miR-29b overexpression also downregulated the expression of the DNA methyltransferase isoforms DNMT1, DNMT3A, and 3B [383]. The global DNA hypomethylation induced by miR-29b led to reexpression of tumor suppressor genes such as the CDK inhibitor p15^INK4b^ [383]. Altogether, these data propose that targeting Mcl-1 with microRNAs such as miR-29 represents a potential tool to constrict tumor growth of Mcl-1 positive lymphomas.

#### 2.5.3. Effect of Bcl-2 Family Proteins on Intracellular Ca^2+^ Mobilization

GCs release Ca^2+^ from the endoplasmic reticulum into the cytosol, which in turn increases the amount of mitochondrial Ca^2+^. The increase in mitochondrial Ca^2+^ induces cytochrome C release and trigger apoptosis. Elevated expression of calcium-binding proteins S100A8 and S100A9 and of the anti-apoptotic Mcl-1 (myeloid cell leukemia-1) inhibits the free cytosolic Ca^2+^ and mitochondrial Ca^2+^ signals, respectively, thereby imposing GC resistance [287, 365, 389, 390]. Downregulation of S100A8 and S100A9 by the Src kinase inhibitor PP2 sensitized MLL-arranged ALL cells otherwise resistant to prednisolone-induced cell death [389]. Bcl-2 inhibits apoptosis in part by decreasing the size of Ca^2+^ stores in the endoplasmic reticulum resulting in reduced Ca^2+^ transfer to the mitochondria [391–393]. One mechanism is through interaction of Bcl-2 with IP_3_R (inositol 1,4,5-triphosphate (InsP3) receptor), which is the principle ER Ca^2+^ release channel in most cell types [394]. Also, Bcl-X_L_ and Mcl-1 act in part by inhibiting IP_3_R [393, 395, 396]. Bcl-X_L_ overexpression also leads to reduced expression of IP_3_R [397].

### 2.6. Presence of Reactive Oxygen Species (ROS) Scavengers

An increase in hydrogen peroxide (H_2_O_2_) is a necessary signal for GC-induced apoptosis [276]. The mitochondria is the source of this signal [275], GCs inhibit complex I and complex III of the electron transport chain [275]. Expression of anti-oxidant defense proteins such as manganese superoxide dismutase, thioredoxin, and catalase prevents GC-induced apoptosis [276, 398–400]. The anti-apoptotic Bcl-2 may regulate the mitochondrial redox state in cancer cells [323, 401].

### 2.7. Increased Notch Activity

Notch is frequently activated in T-ALL cells, which may be due to mutations in Notch1 (gain-of-function) and/or in the E3 ligase Fbw7 that targets Notch1 for degradation [76–78, 80, 81, 402–405]. Some other E3 ligases also regulate Notch signaling [406, 407]. For example LNX1 (ligand of Numb-protein X1) is a positive regulator of Notch signaling through degradation of Numb, a membrane-associated protein that inhibits the function of the Notch receptor [408]. Neuralized (*neur*) and Mind bomb (*mib*) promote the monoubiquitination and endocytosis of Delta [409, 410]. Itch binds to the N-terminal portion of the Notch intracellular domain via its WW domains and promotes ubiquitination of ICN-Notch1 through its HECT ubiquitin ligase domain [411]. Recent studies showed that Notch1 can be activated in leukemic cells through interaction with bone marrow stromal cells that express Notch receptors and ligands [412, 413]. Interaction with bone marrow stroma is also a mechanism for Notch activation in multiple myeloma [414]. The simultaneous expression of Bcl-2 may enforce Notch activity [332, 333]. Cyclin E, which is targeted for degradation by Fbw7 [415, 416], is expressed at higher levels in early relapsed pediatric B-cell precursor ALL patients, who usually show an unfavorable prognosis [143].

Notch1 prevents GC-induced apoptosis, among others, through activation of p56^Lck^, which activates the PI3K-Akt axis [68], and through the transactivation of its target genes *Deltex* and *Hes1* [88]. Hes1 leads to downregulation of PTEN, thereby activating the PI3K/Akt pathway [88]. Deltex is a RING-domain ubiquitin ligase that may affect Notch activity [417], and its overexpression prevents GC-induced apoptosis [418]. Activation of the pro-survival PI3K/Akt/mTOR pathway by Notch has also been observed in other studies [95, 106, 419, 420] and may be responsible for Notch-mediated inhibition of the p53 tumor suppressor gene [95]. Another mechanism by which Notch1 protects T-ALL cells from GC-induced apoptosis, is through the anti-apoptotic GIMAP5/IAN5 (GTPase of the immunity-associated protein/immune-associated nucleotide-binding protein 5) [421, 422]. GIAMP5/IAN5 interacts with Bcl-2 and Bcl-X_L_ and inhibits apoptosis during T-cell development [423] and is highly expressed in human B-cell lymphoid malignancies [424]. It is localized within the mitochondria and endoplasmic reticulum (ER) and regulates mitochondrial integrity [425]. GIMAP has been linked to immunological diseases such as T-cell lymphopenia and autoimmune diseases [426]. Notch also activates NF*κ*B signaling [74, 427] and induces c-Myc expression [428–430], both contributing to apoptotic resistance. Long-term treatment with GCs can overcome Notch1 resistance [67]. This resistance can be overcome by the simultaneous exposure of the cells to Src inhibitors, PI3K/Akt inhibitors, or mTOR inhibitors [67, 68], understating the importance of the protein kinase network in regulating the effects of Notch1 on GC-induced apoptosis. 

A recent report showed that GC sensitivity of T-ALL is associated with GR-mediated inhibition of Notch1 expression [431]. The serum- and glucocorticoid-inducible kinase 1 (SGK1) was also shown to control Notch1 signaling by downregulating its protein stability through Fbw7 ubiquitin ligase [432]. SGK1 phosphorylates Fbw7 at Ser227, an effect inducing ICN-Notch1 ubiquitination and degradation [432]. Despite GC resistance induced by Notch, Notch- and Fbw7-mutated T-ALL shows in general a favorable response to GC therapy and in some studies, but not all, also exhibits a better prognosis [405, 433–436]. This may be related to the fact that GCs may overcome Notch-dependent drug resistance, and in these T-ALL cases the cell survival depends on Notch signaling.

#### 2.7.1. Regulation of Notch Activity by MicroRNAs

Notch activity may be affected by microRNAs [437]. Various microRNAs negatively regulate Fbw7 expression including miR-27a, miR-182, miR-363~92, and miR-223 [253, 438, 439] and may increase the expression of Fbw7-regulated target genes including Notch1, Mcl-1, c-Jun, c-Myc, and Cyclin E [438]. miR-451 and miR-709 suppressed oncogenesis in Notch1-induced mouse T-ALL [440]. miR-150, which is upregulated upon thymocyte maturation, targets Notch3 and thus regulates T-cell proliferation and survival [441]. miR-326 acts in a feedback loop with Notch signaling [442]. The p53-induced miR-34a also targets the Notch1 receptor as well as its ligand DLL1 (Delta like-1) [443, 444].

Prevention of Notch activation in cutaneous T-cell lymphoma (CTLC) by GSI (*γ*-secretase inhibitor) treatment led to alterations in the microRNA profile of the cell [445]. Among others, miR-27a, miR92b, miR-181a, miR-18a, miR-19b, miR-222, and miR-221 were downregulated, while miR-122 and miR-214 upregulated [445]. miR-27a targets Fbw7/hCDC4 [253, 438, 439], the substrate recognition component of the SCF (Skp1-Cullin-F-box) ubiquitin ligase complex that targets Notch1 for degradation [82]. The repressive effect of miR-27a on Fbw7 mRNA is especially pronounced at the G_2_/M and early G_1_ phases [438]. Thus, GSI may indirectly deregulate Notch1 through the miR-27a-Fbw7 pathway. Other targets of miR-27a includes ZBTB10 (zinc finger and BTB domain containing 10), which acts as a repressor of Sp (specificity proteins) transcription factors and induces G1 arrest, and the Myt-1 kinase, which inhibits the transition through G2-M by enhanced phosphorylation and inactivation of Cdc2 (Cdk1, cyclin-dependent kinase 1) [446]. miR-27a is frequently upregulated in pediatric B-ALL [438]. Upregulation of miR-122 by GSI seems to be mediated by p53 and has an antagonistic effect on apoptosis through activation of Akt [85].

### 2.8. c-Myc Overexpression

c-Myc is, among others, a target of Notch [428–430] and has broad effects on tumorigenesis [447] and modulates GC-induced apoptosis [99, 448]. Conditional overexpression of c-Myc in hematopoietic cells in mice culminated in the formation of malignant T-cell lymphomas and acute myeloid leukemias [449]. c-Myc may also be activated in T-ALL independently of Notch1 [450]. These authors demonstrated a role for the PI3K/Akt axis in c-Myc activation. Dysregulation of the c-Myc gene is a common trait of Burkitt's lymphoma due to chromosomal translocations, the most frequent one being t(8; 14)(q24:q32) involving c-Myc and IgH (Immunoglobulin heavy locus) [451–453]. Other hematopoietic malignancies characterized with c-Myc overexpression include diffuse large B-cell lymphoma (DLBCL), follicular lymphoma, CLL, B-cell lymphoma, and AML [454–459]. Earlier studies have shown that dexamethasone-induced apoptosis of a T-ALL cell line was associated with c-Myc suppression [460, 461]. The GC-mediated down-regulation of c-Myc expression was initially thought to be one mechanism that contributes to apoptosis. Not all studies have confirmed this finding [462], which may be explained by the many signaling pathways induced by GCs.

#### 2.8.1. The c-Myc-E2F1-MicroRNA Network

**Figure 4:**
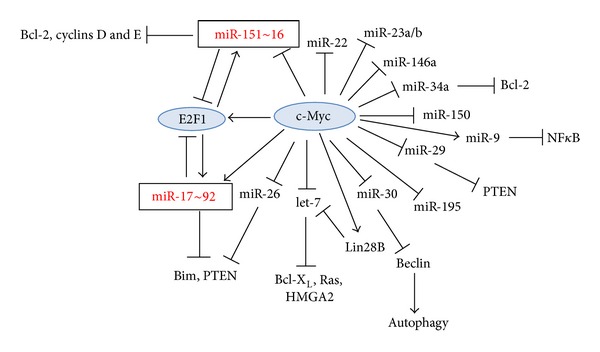
Interplay between microRNAs, c-Myc, and E2F1. Details are described in Section 2.8.1.

c-Myc uses distinct mechanisms for activating and repressing gene expression. For transcriptional activation, c-Myc dimerizes with Max and binds to the promoters of its target genes [463–465]. Transcriptional repression is achieved through protein-protein interactions, where it antagonizes the activity of positive regulators of transcriptions [466]. c-Myc also regulates gene expression by regulating microRNA transcription [255]. The c-Myc-mediated upregulation of miR-17 and miR-20a (belonging to the miR-17~92 cluster) negatively regulates E2F1 translation by targeting the 3-UTR of E2F1 mRNA and may therefore fine tune the direct Myc-mediated transcriptional activation of E2F1, allowing a tightly regulated proliferative signal [255] (Figure 4). E2F1-3 also binds to the promoter of the miR-17~92 cluster and activates its transcription, thus generating an autoregulatory feedback loop [467]. Another target of the miR-17~92 cluster is cyclin D1, which also induces the expression of miR-17 and miR-20a by binding to the promoter regulatory region of the miR-17~92 cluster [468]. The miR-17~92 cluster prevents c-Myc-induced apoptosis [469]. The GC-induced down-regulation of miR-17~92 [206] should actually stimulate E2F1 expression, which under certain circumstances may exert pro-apoptotic effects [470]. E2F1 may promote apoptosis through transcriptional activation of the pro-apoptotic miR-15a~16 cluster [471] and by activating JNK [472]. In a B-cell lymphoma model, c-Myc down-regulated a series of microRNAs, an action that may contribute to tumorigenesis [473]. The c-Myc mediated repression of the miR-30 cluster [473] may affect autophagy, as Beclin-1 expression is regulated by miR-30a [474]. Some of the pro-autophagy activity of cancer therapy is mediated through down-regulation of miR-30a [475]. Also the down-regulation of miR-15a and miR-16 by c-Myc [473] is of interest as these microRNAs are deleted or downregulated in over two-thirds of individuals with CLL, and they target the anti-apoptotic Bcl-2 gene [345, 346]. A third miRNA downregulated by c-Myc is the tumor suppressor let-7 miRNA cluster [473], which targets, among others, the Ras oncogene [476], HMGA2 (high mobility group A2) [477, 478], Bcl-X_L_ [361], Cdc25A, CDK6 (cyclin-dependent kinase 6), and cyclin D2 [479]. Other miRNAs repressed by Myc include miR-22, miR-23a/b, miR-26a/b, miR-29a/b/c, miR-34a, miR-146a, miR-150, and miR-195 [465, 473, 480].

miR-26a levels were found to be reduced in various B-cell lymphomas, especially Burkitt lymphoma [465] as well as various solid tumors [481, 482]. B-CLL, which does not have a prominent pathological role of c-Myc, showed higher expression of miR-26a than Myc-dependent Burkitt lymphoma [465]. miR-26 restoration in Burkitt lymphoma or nasopharyngeal carcinomas reduced proliferation and colony formation through G1 arrest and repression of the histone-lysine N-methyltransferase EZH2, a global regulator of gene expression [465, 481, 483]. The tumor-suppression function was only seen in Myc-transformed cells, but not in v-Abl transformed cells [465, 483]. However, in T-ALL, miR-26a was one of five microRNAs that independently promoted tumorigenesis through inhibition of PTEN [253]. In the background of activating mutations in Notch1, miR-26a overexpression decreased the latency of T-ALL [253].

Forced overexpression of miR-34a, miR-150, and miR-15a/16-1 attenuated in vivo tumor growth of Myc-induced B-cell lymphoma [473]. miR-34a is a crucial component of the p53 tumor suppressor network with potential anti-proliferative and pro-apoptotic activity [484–486]. c-Myc transcriptionally induces Lin28B, which is an RNA-binding protein that suppresses the maturation of let-7 family microRNA precursors [487, 488]. This seems to be one mechanism used by c-Myc to repress let-7 [487]. Lin28 is involved in stem cell maintenance [489–491] and is a marker of cancer stem cells [492].

### 2.9. GC-Induced Autophagy

The effect of autophagy on the cellular response to chemotherapy is dual [493]. Under certain conditions, autophagy acts as a pro-survival mechanism to protect cancer cells from chemotherapy, whereas under other circumstances, autophagy mediates the therapeutic effects of the anticancer agents. Autophagy is regulated by Beclin-1 and autophagy-related genes (ATG) [60]. Another important regulator of autophagy is the activity of mTOR (mammalian target of rapamycin), which is a central element signaling cell growth and enhancing protein translation. When this kinase is inhibited, autophagy is promoted [60].

It should be noted that Beclin-1 may play a dual role in both regulating autophagy and apoptosis, thus being at the cross-road between these two physiological processes. Beclin-1 has recently been recognized as a BH3-only protein interacting with Bcl-2, Bcl-X_L_ and Mcl-1 [59, 60, 494–496]. One report provides evidence that after initiating apoptosis, Beclin-1 is cleaved by caspases and the N-terminal fragment of Beclin can inhibit autophagy, while the C-terminal fragment can amplify mitochondrial-mediated apoptosis [497]. Perturbation of Beclin-1 cleavage by knockin mutation phenocopied the autophagy induction observed in apoptosis-defective cancer cells and rendered chemotherapy resistance both in vitro and in vivo [498]. A role for Beclin in regulating tumorigenesis has been demonstrated in mice with heterozygous disruption of Beclin-1 [499]. These mice have increased frequency of spontaneous malignancies. DLBCL expressing high Beclin-1 levels had a favorable clinical outcome with R-CHOP treatment than those with low Beclin-1 expression [500].

GCs have been shown to promote autophagy in lymphocyte cell lines and primary T-ALL cells [501, 502]. One mechanism for induction of autophagy is through upregulation of the mTOR-inhibitory stress protein Dig2 (dexamethasone-induced gene 2), also known as RTP801 and REDD1 (regulated in development and DNA damage responses 1) [503]. mTOR inhibition by dexamethasone was demonstrated by reduced phosphorylation of S6K (70kD ribosomal protein S6 kinase 1), a member of the RSK family of serine/threonine kinases [503]. Dig2 releases TSC2 from 14-3-3, thereby promoting the assembly of the TSC1/TSC2 complex, which inhibits mTOR [504]. Dig2 knockout thymocytes underwent more extensive dexamethasone-induced cell death, suggesting that autophagy promotes cell survival [503]. However, rapamycin, an inhibitor of mTOR and inducer of autophagy, strongly sensitizes resistant MM and T-ALL cells to GC-induced apoptosis [59, 111, 116, 117], suggesting that induction of autophagy does not always combat apoptosis. It could be that the higher degree of autophagy induced by rapamycin itself may be pro-apoptotic. Bonapace et al. [59] showed that rapamycin induces an autophagy-dependent necroptosis, which is required for childhood T-ALL to overcome GC resistance. Necroptosis is a form of programmed necrosis that occurs when apoptosis is abortive due to caspase inhibition [505]. The GC-mediated necroptosis was mediated by RIP-1 (receptor-interacting protein-1) and CYLD (cylindromatosis) [59]. miR-19, which is frequently overexpressed in T-ALL patients and cell lines, represses CYLD expression [506]. A miR-19 inhibitor induces CYLD expression with consequent decrease in NF*κ*B expression [506]. Obatoclax, a putative antagonist of Bcl-2 family members, could also sensitize T-ALL cells to GC-induced apoptosis through induction of autophagy [59]. This effect was associated with dissociation of the autophagy inducer Beclin-1 from Mcl-1 and decreased mTOR activity [59]. The cell death process could proceed in the absence of Bax and Bak [59]. The apoptosis induced by GC in combination with Obatoclax or rapamycin could be prevented by the autophagy inhibitors 3-methyladenine and bafilomycin [59]. GCs may also induce autophagy by inhibiting Akt activity [501].

### 2.10. Additional Mechanisms Leading to GC Resistance

CDKN2/p16^INK4a^, which acts as a G_0_/G_1_ cycle inhibitor, is frequently lost in T-ALL [507, 508] and predicts relapse in children with ALL [508–510]. p16^INK4a^ sensitizes T-ALL cell lines to GC-induced apoptosis through induction of BBC3/Puma and repression of Mcl-1 and Bcl-2 [511]. Noxa was repressed in p16^INK4a^ transgenic cells, which could be a result of the simultaneous repression of E2F1 due to retinoblastoma protein and p130 activation [511]. The Bim level was unaffected by p16^INK4a^ overexpression [511]. Diffuse large B-cell lymphoma with CDKN2A deletion had a poor prognosis under R-CHOP treatment [512]. Also, *Myc* gene arrangement in diffuse large B-cell lymphoma patients had a poor prognosis with R-CHOP chemotherapy [513].

## 3. MicroRNA in Normal and Malignant ****Lymphoid Cells

During the last decade, microRNAs have become the focus of having a central role in the pathogenesis of cancer including lymphoid malignancies, besides their role in regulating gene expression during cell division, development, and differentiation [514–523]. MicroRNAs are short noncoding RNAs that induce posttranscriptional gene silencing through base pairing with the 3′ untranslated region (UTR) of their target mRNAs, thereby inhibiting their translation, with subsequent reduced protein levels [524, 525]. Bases 2–7 or 2–8 of the microRNA are primary contributors to target specificity and are referred to as the microRNA seed region. The microRNAs are usually transcribed by RNA polymerase II, and sometimes by RNA polymerase III, into long primary precursor transcripts referred to as pri-miRNAs. miRNA are encoded by one arm of a stem loop structure embedded in introns or, less frequently, exons of protein-coding or noncoding transcripts. In the nucleus, the pri-miRNAs stem loop is cleaved by the nuclear RNase III enzyme Drosha together with its cofactor DGCR8 (DiGeorge syndrome critical region 8)/Pasha (the microprocessor complex) to generate ~70 nucleotides long precursors called pre-miRNAs. In some cases, an entire intron consists of such a stem loop structure, which is released by the splicing machinery in a Drosha-independent manner. Such miRNAs are referred to as mirtrons [526, 527]. Pre-miRNAs are exported by RanGTP/exportin-5 to the cytoplasm, where they are further processed by Dicer, another RNase III enzyme, to generate ~22 base pair microRNA duplexes that enter effector complexes called miRISC (miRNA-containing RNA-induced silencing complex). Here, they are converted into single-stranded mature miRNAs that target mRNAs and thereby affect their translation and stability [516, 528, 529].

**Figure 5:**
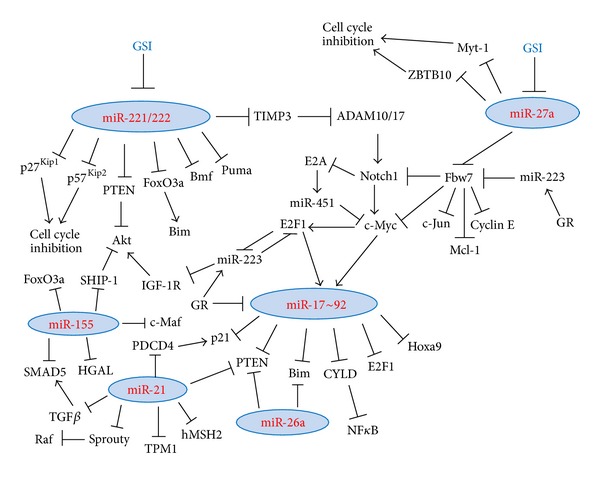
A network of oncomiRs expressed in lymphoid malignancies. A summary of details described in Section 3.

Cancer cells frequently display reduced levels of microRNAs that act as tumor suppressors, while expressing elevated levels of oncogenic microRNAs, called “oncomiRs” that promote tumor development by negatively regulating tumor suppressor genes and/or genes that control cell differentiation and apoptosis. A network of oncomiRs expressed in lymphoid malignancies is depicted in Figure 5. Below I will describe briefly prominent microRNAs detected in normal and malignant lymphoid cells. There are variations in the microRNA expression pattern described between the various scientific reports, which can be explained by the use of different internal standards, different controls for comparison, and the use of sample materials of malignant cells at different developmental stage and at different ontogeny tumor grade.

### 3.1. MicroRNAs in T- and B-Cell Development

Virtually every step in hematopoiesis seems to be finely tuned by specific microRNAs [514, 530–533]. Dicer has an essential role in the development of the adaptive immune system. Conditional deletion of Dicer expression in the T-cell compartment resulted in impaired T-cell development and diminished regulatory T-cell function [534–536], and ablation of Dicer in the B-cell compartment attenuates B-cell development and alters the antibody repertoire [537]. It should be noted that there exists an alternative microRNA processing pathway that is independent of Dicer, but dependent on Argonaute-2 [538].

#### 3.1.1. MicroRNA during Thymocyte Development

MicroRNA expression is dynamically regulated during thymocyte development, with different enriched microRNAs expressed at each developmental stage [539] (Table 1). It should be emphasized that the CD4^+^CD8^+^ (double positive, DP) thymocytes are the most GC-sensitive thymocyte population [540–542]. Dicer-deficient DP thymocytes expressed higher levels of CD69 and TCR (T-cell receptor), but lower levels of Bcl-2 [539]. The Dicer-deficient thymocytes were more prone to apoptosis than control cells [539, 543], understating the role of microRNAs in regulating cell survival. Some microRNAs, such as miR-146a and miR-182, play a dominant role in the regulation of the innate and adaptive immune responses, respectively [544, 545].

According to Neilson et al. [539], the pro-apoptotic miR-15b is almost not expressed at the immature DN1 (double negative 1) thymocyte stage but becomes gradually upregulated in DN3 and DN4, and further in DP cells. The pro-apoptotic miR-16 is also low in DP1 and reaches a maximum in DN4 cells, with a reduction upon transition to DP cells. The oncogenic miR-21 is expressed at the highest level in DN1 and becomes reduced upon transition to DN3 and is almost not expressed in DP cells. miR-181a/b is expressed at the highest level in DP thymocytes, together with miR-92 and miR-350. It should be noted that in this study the expression of each microRNA was determined relative to the general microRNA pool of each subpopulation. Since the amount of total microRNA becomes strongly reduced upon transition from DN4 to DP (a drop from 32000 to 5200 copies/cell), the absolute microRNA number in each cell population differs, which can be demonstrated by the miR-181a transcript. While miR-181a presents 15.6% of the microRNA in DP cells and 6.7% and 5% in DN3 and DN4, respectively, the numbers of miR-181a copies in these three populations were estimated to be 810 in DP, 1400 in DN3, and 1600 in DN4 [539]. Li et al. [546] showed that miR-181a is expressed at DN1 and becomes upregulated during DN2 and DN3 and then downregulated at DN4. miR-181 is still significantly expressed in DP cells, albeit at a slight lesser extent than in DN4 and becomes downregulated upon differentiation to the SP (single positive) stage [546]. miR-146 is upregulated in CD4^+^ T cells [547].

#### 3.1.2. Differentiation Stage-Specific Expression of MicroRNAs in B Lymphocytes

Malumbres et al. [533] performed an extensive microRNA profiling to identify microRNAs specifically expressed in B-cell subsets during peripheral B-cell differentiation. Notably, miR-18a, miR-28, miR-15a~16-1, and miR-181 are expressed at higher levels in centroblasts (germinal center B lymphocytes) compared with memory B cells, whereas miR-101c, miR-150, miR-29a,b,c, and miR-23a~24 are enriched in memory B cells. miR-17~92, miR-363~106a, and miR25~106b are highly expressed in all B-cell subtypes. The high level of miR-15a~16-1 in germinal center B-cells corresponds with low Bcl-2 expression in these cells. miR-223 is highly expressed in naïve and memory B cells, but not in centroblasts. miR-125b is especially expressed in germinal center B lymphocytes [533].

#### 3.1.3. miR-181a/b in T- and B-Cell Development

miR-181a represses the expression of Bcl-2, CD69, and the T-cell receptor (TCR) *α*-chain [539]. miR-181a augments the strength of TCR signaling and down-regulates several phosphatases including DUSP5, DUSP6, SHP-2, and PTPN22 that regulate the sensitivity of T cells to antigens [546]. The down-regulation of PTPN22 by miR-181a led to elevated phosphorylation of p56^Lck^ at Y394 and the down-regulation of DUSP5/6 to increased ERK activation [546]. The normally high levels of miR-181a maintain T-cell tolerance to self-peptide/MHC molecules, with a reduction in this microRNA increasing the number of self-reactive T cells [548]. Also, dampening miR-181a expression using antagomiR-181a impaired positive selection with about a 70% reduction of mature CD4^+^ SP thymocytes [546]. Thus, miR-181a plays a role in regulating TCR response during T-cell development. Recently, miR-181a-1/b-1, but notmiR-181a-2b-2andmiR-181-c/d, was found to control the development of normal thymic T cells and leukemia cells [549]. EctopicmiR-181a-1expression in thymic progenitor cells potentiated DP cell development[549]. Conditional deletion of miR-181ab1 allele resulted in 50%–75% decrease in cellularity in the thymus and a significant reduction in the percentage of DP cells [549]. miR-181a expression decreased during the DN3a to DN3b transition during *β*-selection, and loss ofmir-181ab1resulted in a reduction in the percentage of DN3 and DN4 cells that expressed intracellular TCR-*β*, while preT*α* expression in DN3 thymocytes was normal [549].

miR-181a becomes downregulated when mouse T cells are costimulated with TCR and CD28 [317]. Other alterations occurring upon TCR/CD28 co-stimulation includes the upregulation of the miR-466 family, miR-574, miR-346, miR-214, miR-155, and miR-709, and the down-regulation of the miR-29 family, miR-15a, miR-15b, miR-16, miR-146b, miR-142, miR-27a, miR-150, and let-7 family [317].

Chen et al. [550] showed that miR-181 is expressed in the B-lymphoid cells of the mouse bone marrow, and its ectopic overexpression in hematopoietic stem/progenitor cells significantly increased B-cell production [550]. miR-181 also affects the development of NK cells through targeting the Nemo-like kinase (NLK), an inhibitor of Notch signaling [551]. miR-181 targets the RNA-binding protein Lin28, thereby disrupting the Lin28-let-7 reciprocal regulatory loop, with concomitant upregulation of let-7 and differentiation of megakaryocytes [552].

#### 3.1.4. miR-150 in T- and B-Cell Development

miR-150 is highly expressed in mature and resting lymphocytes, but not in their progenitors [547, 553]. Overexpression of miR-150 led to a block in B-cell formation at the pro-B to pre-B-cell transition by downregulating c-Myb, among other targets [547], suggesting for a role for this microRNA in B-cell differentiation. Within the lymphoid lineage the choice between T and B cells is regulated by miR-150 [547, 553]. The T-cell population level was unaffected by overexpression of miR-150 in hematopoietic progenitor cells, while the mature B-cell levels were strongly reduced [553]. miR-150 drives megakaryocyte-erythrocyte progenitor (MEP) cells towards megakaryocytes at the expense of erythroid cells [554]. miR-150 also regulates the development of NK (natural killer) and iNKT (invariant NK) cells [555]. Mice with target deletion in miR-150 had a defect in their ability to generate mature NK cells, while overexpression of miR-150 resulted in a substantial reduction in iNKT in the thymus and in the peripheral lymphoid organs [555], supposedly through targeting of c-Myb by miR-150 [556].

#### 3.1.5. miR-125b in T- and B-Cell Development

miR-125b affects T-cell differentiation through regulation of IFN*γ* (Interferon *γ*), IL-2R*β*, IL-10R*α*, and PRDM1/Blimp1 (B lymphocyte-induced maturation protein-1) [592]. Ectopic expression of miR-125b in naïve lymphocytes inhibited differentiation to effector cells [592]. During normal B-cell development, miR-125b is enriched in germinal center B cells and keeps the transcription factor IRF4 and PRDM1/Blimp1 down, while miR-223 is enriched in memory B cells, where it targets the transcription factor LMO2, which is specifically expressed in germinal center B cells [533]. IRF4 and PRDM1/Blimp1 expression are repressed in centroblasts, but is necessary for differentiation into memory and plasma cells [593, 594]. Overexpression of miR-125b alone in mice causes an aggressive, transplantable myeloid leukemia [357]. Before leukemia, these mice did not display elevation of white blood cells in the spleen or bone marrow, rather the hematopoietic compartment showed lineage-skewing, with myeloid cell numbers dramatically increase and B-cell numbers severely diminished [357]. miR-125b targets *Lin28A*, an induced pluripotent stem cell gene [595]. Knockdown of Lin28A led to hematopoietic lineage-skewing similar to ectopic miR-125b overexpression, with increased myeloid and decreased B-cell number [595]. miR-125b is also a potent oncomiR in the development of megakaryoblastic leukemia [596].

#### 3.1.6. miR-155 in T- and B-Cell Development

miR-155 is also important for lymphopoiesis and for preserving normal immune system responses [266, 597–599]. miR-155 is processed within the second exon of the nonprotein-encoding gene *BIC* (B-cell integration cluster). miR-155 is upregulated upon TCR/CD28 costimulation in mouse T cells [317], and in macrophages by several TLR (Toll-like receptor) pathways [600]. B cells require miR-155 for normal production of isotype-switched, high-affinity antibodies and for a memory response [599]. miR-155 knockout mice are immunocomprised owing to defects in B and T lymphocytes [597]. The transcription factor PU.1, which down-regulates IgG1 levels, is a target gene of miR-155 in B cells [599]. This may explain the reduced amount of circulating IgG1 in miR-155 knockout mice [599]. As with B cells, it seems that miR-155 is involved in T-cell differentiation [266, 597]. Naïve T cells derived from miR-155 knockout mice showed increased propensity to differentiate into Th2 rather than Th1 cells, with the concomitant production of Th2 cytokines such as IL-4, IL-5, and IL-10 [266, 597]. One explanation for this biased development of Th2 cells might be the miR-155 mediated targeting of c-Maf (musculoaponeurotic fibrosarcoma), a transcription factor that transactivates the IL-4 gene [597]. With regard to the acute immune response, the T cells had an impaired response and showed attenuated IL-2 and IFN*γ* release in response to antigens [266, 597]. miR-155 is upregulated by the transcription factor FoxP3 and critical for T regulatory cell function [601]. Mice overexpressing miR-155 in the B-cell lineage results in preleukemic pre-B-cell proliferation in the spleen and bone marrow, followed later in life by B-cell malignancy [602]. miR-155 represses genes encoding DNA damage response proteins [603].

#### 3.1.7. miR-17~92 in T- and B-Cell Development

The miR-17~92 cluster located on chromosome 13 at locus q31.3 is essential for B-cell development [246]. The expression of miR-17~92 peaked in pre-B cells, where it inhibited cell death [246]. It is expressed at higher levels in normal germinal center B cells compared to naïve and memory B cells [533]. Knockout of miR-17~92 leads to increased Bim expression and inhibits B-cell development at the pro-B to pre-B transition [246], a step also blocked by miR-150 [553]. Mice overexpressing the miR-17~92 cluster in lymphocytes developed lymphoproliferative disease and autoimmunity and they died prematurely [247]. These animals were found to have increased numbers of activated B cells, and a higher ratio of activated CD4^+^ T cells versus CD8^+^ T cells. The enhanced proliferation and survival of B and T cells may result from the down-regulation of Bim and PTEN [247]. miR-17~92 expression is strongly induced after activation of CD8^+^ T cells, which is critical for the rapid clonal expansion of these cells [604]. However, following the clonal expansion, miR-17~92 is downregulated and further silenced during memory development [604].

### 3.2. MicroRNAs in Lymphoid Malignancies

Malignant lymphomas arise from normal B- or T-cell counterparts at different ontogeny stages and commonly continue to express gene signatures inherited from their nontransformed cellular progenitors. Extensive miRNA profiling studies have been performed on various lymphoid malignancies, including T-ALL [605], cutaneous T-cell lymphoma [606], CLL [557, 563], pre-B-ALL [557, 605, 607], diffuse large B-cell lymphoma (DLBCL) [564, 580–582, 608–610], anaplastic large cell lymphoma (ALCL) [587], multiple myeloma (MM) [574, 611, 612], mantle cell lymphoma (MCL) [583, 591, 613], Burkitt Lymphoma (BL) [564, 614], and follicular lymphoma (FL) [135, 582, 615]. A comprehensive study aimed to integrate the many miRNAs upregulated in T-ALL into a microRNA-transcription factor coregulatory network was performed by Ye et al. [506]. Various microRNAs have also been associated with poor prognosis [515]. A short description of some important microRNAs in malignant lymphoid diseases is described below and summarized in Tables 2 and 3.

#### 3.2.1. MicroRNAs in T-Acute Lymphoblastic Leukemia (T-ALL)

In general, T-ALL is characterized by upregulation of the miR-17~92 cluster, miR-26a, miR-128a/b, miR-146a, miR-181a/b, miR-150, and miR-155, while let-7b and miR-223 are downregulated [253, 557–561].

*3.2.1.1. miR-17 *~*92 in T-ALL*. The miR-19, miR-20a, miR-92a, and miR-17 especially of the miR-17~92 cluster are upregulated in T-ALL [506]. All six miRNAs miR-17, miR-18a, miR-19a, miR-20a, miR-19b, and miR-92a, of the miR-17~92 cluster promoted leukemogenesis in Notch1-induced T-ALL in vivo [253, 616]. Among them, the miR-19 family has been considered the key oncogenic component [248, 506, 617]. The miR-17~92 cluster is located within a fragile site that is frequently amplified in a range of hematopoietic malignancies [618]. Paralogues to the miR-17~92 cluster include miR-106b~25 and miR-106a~363 [246, 619].

miR-19 represses Notch1, PTEN, Hoxa9, Cyld, Runx1, E2F1, and Bcl2L11 (Bim) [506, 616, 620]. Reduced expression of Bim attenuates GC-induced apoptosis. Posttranslational inactivation of PTEN by miR-19 promotes activation of the PI3K/Akt pathway, and incontrollable proliferation of T cells [104, 105]. Increased Akt signaling antagonizes GC-induced apoptosis by several mechanisms, including phosphorylation of FoxO3a, thus preventing its nuclear translocation and transcriptional activation of Bim, and through inactivation of GSK3, which is essential for GC-induced apoptosis [30, 67, 97].

Hoxa9 is a leukemogenic homeoprotein in T-ALL [621], and a target gene of the oncogenic MLL-AF4 fusion protein [622]. High expression of miR-196b was found in pediatric ALL with aberrant activation of Hoxa genes [623].

Notch1 plays a vital role in T-cell development and transformation, and about 50% of primary T-ALL samples show abnormal Notch1 expression [79]. Downstream transcriptional targets of Notch1 include *Hes1* and c-Myc, the former affecting the PI3K/Akt and NF*κ*B signaling pathways [624, 625]. c-Myc is a potent and direct transcriptional activator of miR-17~92, leading to modulation of E2F1 expression [255]. Deletion of miR-17~92 cluster repressed Myc-induced oncogenesis [246, 248]. GCs repress the expression of miR-17~92 [206], which may be one means to overcome the tumorigenicity of T-ALL cells and to elevate Bim expression [206].

In contrast to miR-19, miR-451, and miR-709 are potent suppressors of oncogenesis in Notch1-induced mouse T-ALL [440]. miR-451 represses c-Myc, while miR-709 represses Ras-GRF-1 that acts upstream to Ras and prevents Akt activation [625]. Both miR-451 and miR-709 are transcriptional targets of the bHLH E2A tumor suppressor, which is degraded upon Notch1 induction in mouse T-ALL cells [626, 627]. Repression of tumor suppressor miR-451 is essential for Notch1-induced oncogenesis in a murine model of T-ALL [440]. Human T-ALLs with activating Notch1 mutations have decreased miR-451 and increased c-Myc levels compared with T-ALLs with wild-type Notch1 [440]. One mechanism of the tumor suppressive action of miR-451 could be through down-regulation of the PI3K/Akt survival pathway [628]. 

*3.2.1.2. miR-26a in T-ALL*. Primary T-ALL cells also express elevated levels of miR-26a that suppresses PTEN and Bim [253]. miR-26a enhanced leukemogenesis in a mouse model of T-ALL [253]. miR-26a was found to be repressed by c-Myc in a mouse lymphoma model, leading to enhanced expression of the EZH2 oncogene, a component of the Polycomb repressive complex 2 [483]. c-Myc may also directly upregulate EZH2 [629]. In mantle cell lymphoma, miR-26a was found to affect NF*κ*B nuclear translocation [591]. 

*3.2.1.3. miR-146a in T-ALL*. miR-146a, miR-181a/c, and miR-221 were associated with overall survival in ALL patients [559]. miR-146 seems to have opposing roles in tumorigenesis depending on the cellular context [517]. miR-146a and miR-146b are elevated in several types of solid tumor [630–633]. However, overexpression studies of miR-146a in transplanted bone marrow cells suggest a tumor-suppressive role for this microRNA [634]. miR-146 overexpression reduced the survival and engulfment of hematopoietic stem cells in recipient cells [634]. miR-146a knockout mice developed massive myeloproliferation followed by hematopoietic tumors, including myeloid sarcomas and lymphomas [635, 636]. The myeloproliferative phenotype correlated with enhanced NF*κ*B signaling [636]. miR-146a suppresses the NF*κ*B activators IRAK1 (interleukin 1 receptor-associated kinase 1) and TRAF6 (TNF receptor-associated factor 6) [635, 637, 638]. Thereby, overexpression of miR-146a leads to inhibition of NF*κ*B activity. A negative feedback loop exists between NF*κ*B and miR-146. Whereas miR-146 represses NF*κ*B signaling, NF*κ*B signaling upregulates miR-146 [637]. 

*3.2.1.4. miR-181a in T-ALL*. miR-181a family members are highly expressed in T-ALL leukemia cells and downregulated during remission[639]. Deletion ofmiR-181a-1/b-1expression inhibits the development ofNotch1 oncogene-induced T-ALL in a mouse model [549]. miR-181a/bcontrols the strength and threshold of Notch activity in tumorigenesis in part by dampening multiple negative feedback regulators downstream of Notch and pre-T-cell receptor (TCR) signaling pathways [549]. 

*3.2.1.5. miR-124a in T-ALL*. miR-124a has been shown to be downregulated in more than 50% of ALL cases and associated with higher relapse rate and mortality rate [560]. It targets CDK6 and reduces Rb (retinoblastoma) phosphorylation. Its down-regulation contributes to the abnormal proliferation of ALL. Inhibition of CDK6 by sodium butyrate or PD0332991 decreased ALL cell growth. Overexpression of miR-124a reduced tumorigenicity in a xenogeneic mouse model [560].

#### 3.2.2. MicroRNAs in Chronic Lymphocytic Leukemia (CLL)

A comparison study of primary CLL samples with normal unstimulated or CpG-stimulated B cells showed high similarities between CLL and activated B cells, including upregulation of miR-34a, miR-155, and miR-342 and down-regulation of miR-103 and miR-181a/b [565]. Activation of normal B cells led to reduced miR-23a, miR-23b, miR-24, miR-27b, miR-181a/b, and miR-223 and increased miR-155 with all activation agents used. Differential effect on miR-29 family was observed with the different activation agents. One particular difference between activated B cells and CLL was seen with miR-150. miR-150 was reduced during B-cell activation, whereas it was upregulated in most CLL cases [565]. The latter confirms the study of Fulci et al. [566], but is opposed to Wang et al. [682] showing that miR-150 is downregulated in CLL. Ectopic miR-150 expression increased cell death in pro-B cells, while miR-150 deficiency led to B-cell expansion and an enhanced humoral immune response [547]. Some differences in miR-150 are observed between the mutated versus unmutated IgV_H_ (immunoglobulin heavy chain variable-region genes) subgroups, where expression is higher at the average in the mutated IgV_H_ subgroup [566].

CLL cases with unmutated IgV_H_ or with high expression levels of ZAP-70 (70kD zeta-associated protein) show an unfavorable course with rapid progression in comparison to patients with a mutated IgV_H_ [567]. Two research groups [565, 566] observed decreased levels of miR-29c and miR-223 in CLL with ZAP70^+^ and IgV_H_ unmutated status. Calin et al. [643] observed that the unmutated IgV_H_ CLL subgroup exhibited high levels of Tcl-1 due to low expression of miR-29 and miR-181 that negatively regulate this oncogene. miR-181 and miR-29 might therefore be considered to have tumor-suppressor functions. Tcl-1 functions as a coactivator of Akt [707], and B-cell forced expression of Tcl-1 in transgenic mice resulted in tumors that resembled CLL [567]. CLL with unmutated IgV_H_ and high expression of ZAP-70 showed also relative high expression of miR-15a, miR-16-1, miR-16-2, miR-195, miR-23b, miR-155, miR24-1, and miR-146, while low expression of miR-223, miR-29a-2, miR-29b-2, and miR-29c [563]. In an aggressive subtype of CLL with abnormalities in the *TP53* gene, the microRNAs miR-34a, miR-29c, and miR-17 were downregulated [567]. 

*3.2.2.1. miR-15a~16 in CLL*. CLL cases with good prognostic features are typically characterized by down-regulation of miR-15a and miR-16-1 [643, 708], located at the 13q14.3 locus. These miRNAs map to a region between exon 2 and 5 of the *Leu2* gene. Deletion of 13q14.3 (del(13q)) is the most common cytogenetic abnormality in CLL occurring in more than 50% of the cases and implies for a favorable prognosis [709]. This deletion occurs also frequently in MM patients [575].Deletion in mice of the 13q14-minimal deleted region, which encompasses the miR-15a~16 cluster, caused the development of indolent B-cell-autonomous, clonal lymphoproliferative disorders, recapitulating the spectrum of CLL-associated phenotypes observed in humans [644]. Repression of miR-15a and miR-16-1, as well as miR-29b, in CLL may also be mediated by histone deacetylases (HDACs) [710]. HDAC inhibition triggered the accumulation of the transcriptionally activating chromatin modification H3K4me2 and restored the expression of miR-15a, miR-16-1, and miR-29b [710]. Deacetylase inhibition may therefore be an attractive therapeutic strategy.

Both miR-15a and miR-16-1 negatively regulate Bcl-2 [643], and miR-29 targets Mcl-1 [381, 382]. The expression of Bcl-2 in CLL cases is inversely correlated with the expression of miR-15a and miR-16-1 [563, 711]. Other targets of miR-15/16 include CHEK1 [615], CyclinD1, CyclinD2, and Cdc25A [525, 645]. Overexpression of miR-15a and miR-16-1 induced cell cycle arrest at G1/G0 in an Rb-dependent manner [712]. A germ-line mutation in the primary precursor of miR-15a/16-1 that impairs their processing was observed in familial CLL patients [563]. Targeting deletion of miR-15a~16 in mice led to the development of a spectrum of diseases resembling CLL-associated lymphoproliferation in humans, including CLL, CD5^+^ monoclonal B-cell lymphocytosis, and CD5^−^ non-Hodgkin's lymphomas [644]. The New Zealand black (NZB) mouse that harbor a point mutation in the 3′-flanking region of miR-16 that leads to reduced miR-16 expression and develops symptoms similar to B-CLL in humans, further confirming the tumor suppressor function of this locus [713]. 

*3.2.2.2. miR-181a/b in CLL*. Underexpression of miR-181a/b was associated with shorter overall survival in CLL [358], while higher levels of miR-181a were associated with a shorter time from diagnosis to initial therapy [563]. During the course of CLL progression, the miR-181a/b levels were decreased, which inversely correlated with increased levels of its target genes *Mcl-1* and *Bcl-2* [385]. miR-181b was especially downregulated in treatment-refractory cases [714]. The study of Marton et al. [568] showed consistent underexpression of miR-181a, as well as let-7a and miR-30d in all CLL cases studied. However, increased expression of miR-181a/b was associated with favorable outcome in patients with cytogenetically normal acute myeloid leukemia (AML) [692].

Ectopic overexpression of miR-181a/b into primary CLL increased fludarabine-sensitivity in p53 wild-type cells, but not in CLL with attenuated p53 response [358]. The importance of the miR-181 target Mcl-1 in CLL survival was demonstrated by rapid apoptosis of CLL cells following siRNA-mediated down-regulation of Mcl-1 [715], and by the Mcl-1 transgenic mice, which developed B-cell lymphoma [715]. Thus, low miR-181 and miR-29 expression in CLL could confer drug resistance through upregulation of Mcl-1 expression. 

*3.2.2.3. miR-29 in CLL and Other B-Cell Malignancies*. The miR-29 family consisting of miR-29a and miR-29b seems to play a dual role in tumorigenesis [517]. On the one hand, miR-29a and miR-29b are downregulated in mantle cell lymphoma [583], aggressive CLL samples (high ZAP-70 with unmutated IgV_H_) [659, 710, 716], ALK-positive anaplastic large cell lymphomas (ALCL) [380], MM [381], and AML [383]. On the other hand, miR-29a and b are expressed at higher degree in indolent CLL (low ZAP-70/mutated IgV_H_) than in normal CD19^+^ cells [563, 569, 716]. miR-29c together with miR-223 down-regulation is associated with higher tumor burden, disease aggressiveness, and poor prognosis in CLL [700].

Forced overexpression of miR-29b induced apoptosis in MM and AML cells [381, 383]. The tumor suppressor activity of miR-29 may be achieved through targeting cell cycle regulators and oncogenes such as Cdk6, DNA methyltransferase 3A (DNM3A) and 3B (DNMT3B), Mcl-1, and Tcl1A [382, 569, 583, 717]. Another tumor suppressor function of miR-29 is mediated through activation of p53, which is achieved by targeting p85*α* (the regulatory subunit of PI3K kinase) and CDC42 (a Rho family GTPase) [660].

However, in another setting miR-29 acts as an oncogene. miR29a overexpression in immature and mature B cells promoted CLL development [716], and transplantation of miR-29-transduced hematopoietic stem and progenitor cells into irradiated mice resulted in myeloproliferative disease and AML [661]. One mechanism for the oncogenic feature of miR-29 could be through repression of the tumor suppressor cell-adhesion molecule peroxidasin homologue (PXDN) [716]. Thus, depending on the cellular contexts,miR-29 can function as an oncogene or a tumor suppressor. 

*3.2.2.4. miR-221/222 in CLL*. miR-221 and miR-222 are expressed at higher levels in CLL with unmutated IgV_H_ and high expression of ZAP-70, the most aggressive CLL subtype with poor prognosis [563]. These microRNAs may contribute to oncogenesis by targeting the CDK inhibitor p27^Kip1^ [695, 696, 718, 719], FoxO3a [720, 721], Apaf-1 [721, 722], p57^Kip2^ [719], Bmf [723], PTEN [321], and TIMP3 (tissue inhibitor of metalloproteinase 3) [321]. In other CLL cases, the miR-222 was found to be lower than that of normal CD19^+^ cells [566]. miR-221 was expressed at reduced levels in CLL harboring the 13q14 deletion [711]. 

*3.2.2.5. miR-34 in CLL*. The p53 target miR-34a is decreased in CLL patients with 11q deletions, leading to increased ZAP-70 expression [663]. miR-34a also targets Bcl-2 [348, 484], and the E2F1 and B-Myb oncogenes in CLL [664]. Reduced miR-34a expression has been associated with resistance to DNA damage in CLL [570]. 

*3.2.2.6. miR-17~92 in CLL*. Members of the miR-17~92 polycistron are upregulated in B-cell lymphoma, as well as miR-155 [469, 568, 685]. Adoptive transfer of hematopoietic stem cells bearing a truncated portion of the miR-17~92 polycistron in c-Myc transgenic mice resulted in a more rapid onset of malignant B-cell lymphomas. These lymphomas exhibited resistance to apoptosis and increased proliferation [469]. Transgenic overexpression of the entire miR-17~92 in the murine hematopoietic compartment led to the development of lymphoproliferative disease and increased lethality [247]. The negative regulation of Bim by the miR-17~92 cluster seems to be a major mechanism by which B-cell lymphomas evade apoptosis [247]. Silencing of miR-17 and miR-20a in mantle cell lymphoma led to upregulation of the cyclin-dependent kinase (CDK) inhibitor p21, suggesting that p21 is an essential target of the miR-17~92 cluster during B-cell lymphomagenesis [647]. Overexpression of c-Myc mRNA together with miR-17-5p/miR-20a was associated with a more aggressive behavior in mantle cell lymphoma [724]. miR-17~92 confers chemoresistance in mantle cell lymphoma through activation of the PI3K/Akt pathway [725]. Knockdown of miR-17~92 inhibited tumor growth in a xenograft mantle cell lymphoma model [725]. 

*3.2.2.7. miR-21 in CLL*. miR-21 is commonly upregulated in CLL [650] as well as CML [726] and many other cancer cell types [525]. Forced overexpression of miR-21 under the control of the nestin promoter resulted in severe pre-B-cell lymphoma [727]. miR-21 overexpression potentiated lung tumorigenesis of a constitutively activated K-Ras proto-oncogene [728]. miR-21 deletion in mice reduced 7,12-dimethylbenz[a]anthracene (DMBA)/12-O-tetradecanoylphorbol-13-acetate(TPA) skin carcinogenesis [729]. miR-21-null mice exhibited an increase in cellular apoptosis and decrease in cell proliferation [729]. miR-21 is an oncomiR that promotes tumorigenesis by targeting a range of genes involved in regulating cell proliferation and/or survival, including PTEN [269], Sprouty (Spry2) [730], PDCD4 (programmed cell death 4) [731], TPM1 (tropomyosin 1) [651], and human DNA MutS homolog 2 (hMSH2) [732]. In glioblastoma cells, miR-21 also targets a network of p53 pathways, TGF*β*, and mitochondrial tumor suppressor genes [733]. PDCD4 inhibits AP-1-mediated transactivation [734] and negatively regulates the pro-survival RAL guanine-nucleotide dissociation stimulator (RALGDS) signaling pathways [517, 729]. PDCD4 also induces the expression of the CDK inhibitor p21 [735]. Down-regulation of PDCD4 by miR-21 confers growth advantages to the cells. PDCD4 is a tumor suppressor that is upregulated during apoptosis [736] and downregulated in several cancer forms [737–739]. Spouty, which is downregulated by miR-21, negatively regulates the c-Raf pro-survival signaling pathway [729]. 

*3.2.2.8. miR-125b in CLL*. Both aggressive and indolent CLL patients showed reduced expression of miR-125b [571]. Overexpression of miR-125b in CLL-derived cell lines resulted in the repression of many transcripts encoding enzymes implicated in cell metabolism [571]. These authors proposed that miR-125b acts as a regulator for the adaptation of cell metabolism to a transformed state. 

*3.2.2.9. miR-150 in CLL*. One microRNA consistently downregulated in most B-lymphomas is miR-150 [682], which is proposed to act as a tumor suppressor [523, 547, 589]. Mice lacking miR-150 have increased expression of its target transcription factor c-Myb, which plays an important role in lymphocyte development and maturation [547]. miR-150 is especially expressed in mature lymphocytes, but not in their progenitors [547]. Premature expression of miR-150 blocked the transition from pro-B to the pre-B stage [553]. Overexpression of miR-150 in NK/T lymphomas increased apoptosis and reduced cell proliferation, with concomitant reduction in DKC1 (Dyskeratosis congenita 1) and Akt2, reduced Akt phosphorylation, and elevated levels of Bim and p53 [683]. 

*3.2.2.10. miR-155 in CLL*. miR-155 is overexpressed in many B-cell lymphomas including CLL, primary mediastinal B-cell lymphoma (PMBL), aggressive activated B-cell like (ABC) subtype of DLBCL, Hodgkin's lymphoma, and pediatric Burkitt's lymphoma, but is almost absent in adult Burkitt's lymphoma [515, 566, 568, 576, 583, 584, 587, 602, 685, 686]. c-Myb (v-Myb myeloblastosis viral oncogene homolog), which is overexpressed in a subset of CLL patients, associates with the promoter of miR-155 host genes (miR155HG, also known as BIC, B-cell integration cluster) and stimulates its transcription [687]. Forced overexpression of miR-155 in B cells (E*μ*-miR-155 transgenic mice) led to initial preleukemic pre-B-cell proliferation followed by frank B-cell malignancy [602]. The miR-155 orthologue miR-K12-11 in Kaposi sarcoma-associated herpes virus (KSHV) has been associated with B-cell tumors [740]. miR-155 is essential for immune function and is strongly induced in activated T and B cells [597]. miR-155 represses SH2-domain containing inositol-5-phosphatase-1 (SHIP-1), which is a critical phosphatase that negatively downmodulates Akt pathway and is involved in normal B cell development [688]. Thus, sustained overexpression of miR-155 in B cells unblocks Akt activity, inducing B-cell development. miR-155 targets c-Maf in lymphocytes [597], and HGAL and SMAD5 in diffuse large B-cell lymphoma (DLBCL) [741, 742]. *HGAL*, a germinal center (GC)-specific gene, inhibits lymphocyte and lymphoma cell motility by activating RhoA signaling cascade [743] and by interacting with actin and myosin proteins [744]. SMAD5 is a bone morphogenetic protein (BMP)-responsive transcription factor and is activated by various cytokines [745]. DLBCL expressing high levels of miR-155 concomitant with low HGAL expression showed high aggressiveness and cell dissemination [741]. siRNA-based SMAD5 knockdown recapitulated the effects of miR-155 overexpression in DLBCL [742]. Thus, down-regulation of SMAD5 in diffuse large B-cell lymphoma defines a unique mechanism used by the cancerous cells to escape TGF*β* growth inhibitory effects [742]. In breast cancer, miR-155 targeted FoxO3a, thus modulating their response to chemotherapy [264]. As FoxO3a is a positive regulator of the pro-apoptotic Bim essential for GC-induced apoptosis [227–229], miR-155 overexpression may prevent Bim upregulation.

#### 3.2.3. miRNAs in Multiple Myeloma (MM)

In one study, miR-93, miR-25, miR-92, miR-19a/b, miR-181a/b, and miR-32 were shown to be significantly overexpressed, while let7-b, let7-I, let7-c, miR-29a, and -29b significantly downregulated in MM [574]. Roccaro et al. [575] found decreased expression of miR-15a~16 and increased expression of miR-222, miR-221, miR-382, and miR-181a/b in their MM samples. Heterogeneous expression of miR-181a and -181b was observed in MM cells from many patients [574]. Also, the 13q14.3 locus containing the miR-15a and miR-16-1 is sometimes deleted in MM [345, 746–748]. The absence of miR-15a expression and overexpression of miR-181a/b correlated with worse prognosis of MM [575]. Antagonists especially to miR-19a/b and miR-181a/b (AntagomiRs) suppressed tumor growth of human myeloma cells implanted into nude mice [574]. This finding demonstrates the potential use of microRNAs in therapy.

Some differential miRNA expression was observed between malignant MM and MGUS (monoclonal gammopathy of undetermined significance) [574], which is the precancerous state preceding MM [749]. MGUS show already upregulation of miR-21, miR-106~25, miR-181a/b, miR-1, and miR-133a, while during the progression to malignant multiple myeloma miR-17~92, miR-32, miR-193b~365 are upregulated and miR-192~194~215 and miR-15a~16 are downregulated [574, 576, 577]. The upregulation of miR-17~92 could be related to the upregulation of c-Myc observed during MM progression [750, 751]. Upregulation of miR-1 and miR-133a correlated with t(14; 16) translocation in MM cases, suggesting that deregulation of microRNA expression could be associated with chromosomal abberations [578]. MGUS premalignant cases displayed higher levels of Dicer than MM cells [752]. Higher expression of Dicer was associated with improved progression-free survival in symptomatic MM cases [752].

The global increase in microRNA expression in high-risk MM patients with poor prognosis was associated with increased expression of Argonaute (AGO2/ElF2C2) [611], a master regulator of miRNA maturation and function [753, 754]. Silencing of AGO2 decreased viability in MM cell lines [611]. 

*3.2.3.1. IL-6 and MM*. Adhesion of multiple myeloma to bone marrow stroma triggers cytokine production and enhances cell proliferation and resistance to chemotherapy through IL-6-induced activation of NF*κ*B, PI3K/Akt, and STAT3 pathways [755]. It should be noted that these pro-survival pathways antagonize GC-induced apoptosis in MM [756–760]. miR-19a and miR-19b that are part of the miR-17~92 cluster downregulate *SOCS-1* (suppressor of cytokine signaling-1), a gene frequently silenced in MM that plays a critical role as inhibitor of IL-6 growth signaling [574], thus enforcing the IL-6-induced survival signals. 

*3.2.3.2. miR-21 in MM*. The oncogenic miR-21 is upregulated in MM patient samples and cell lines [574, 579, 652]. In IL-6-dependent MM cell lines, miR-21 transcription is controlled by IL-6 through a STAT-3 mechanism. Ectopic miR-21 expression was sufficient to sustain growth of IL-6-dependent cell lines in the absence of IL-6 [761]. miR-21 is upregulated in a NF*κ*B-dependent manner in MM cells upon cell adhesion to bone marrow stromal cells [762]. Combining miR-21 inhibition with dexamethasone inhibited MM cell survival more effectively than either treatment alone [762]. The p300-CBP-associated factor (PCAF) was found to be a target of the combined action of the miR106b~25 cluster and miR-32 [574]. PCAF is a positive regulator of p53 through ubiquitination activity on Hdm2 [763]. miR106b~25, miR-17, and miR-20a target the CDKN1A1/p21 cell cycle regulator, which prevents cell cycle progression in general and prevents the growth of MM cells [764, 765]. 

*3.2.3.3. miR-15a~16 in MM*. miR-15a~16 is a pro-apoptotic microRNA that targets Bcl-2, cyclin D1, cyclin D2, and Cdc25A [346, 748, 766–768]. Overexpression of miR-15a~16 in MM led to inhibition of Akt3, ribosomal protein S6, MAP kinases, and the NF*κ*B-activator MAP3KIP3, ultimately resulting in an antiproliferative effect and apoptosis [575]. The anti-MM effect of miR-15a~16 was observed even in the context of the bone marrow microenvironment [575]. miR-15a~16 reduced VEGF secretion from MM cells, thereby reducing MM cell-induced pro-angiogenic activity on endothelial cells [575]. VEGF represents one of the major pro-angiogenic cytokines responsible for the induction of neoangiogenesis in MM patients [769, 770].

#### 3.2.4. miRNAs in Anaplastic Large Cell Lymphoma (ALCL)

A distinct microRNA profile could distinguish between ALK^+^ and ALK^−^ subtypes of ALCL, an aggressive form of non-Hodgkin's lymphoma (NHL) belonging to the T-cell lineage [587]. More than 80% of ALK^+^ ALCL harbor the t(2; 5)(p23; q35) translocation, resulting in the expression of the chimeric nucleophosmin (NPM)-ALK [771]. The constitutive ALK activity leads to the activation of many different growth-promoting and anti-apoptotic pathways including PI3K/Akt/mTOR, Jak/Stat, c-Jun, JunB, and c-Myc. The prognosis of ALK^−^ ALCL is worse [772, 773]. ALK^+^ ALCL has a high cure rate with CHOP treatment, in contrast to ALK^−^ cells that are relative resistant [774]. Five members of the miR-17~92 cluster were expressed higher in ALK^+^ ALCL, whereas miR-155 was expressed more than 10-fold higher in ALK^−^ ALCL [587]. The upregulation of miR-17~92 cluster in ALK^+^ ALCL cells is in agreement with the observation that c-Myc is expressed in ALK^+^ ALCL and absent from ALK^−^ samples [775]. miR-101 was downregulated in all ALCL tested [587]. miR-101 targets mTOR [776], Mcl-1 [777], and the histone methyltransferase EZH2 [778, 779]. Inhibition of mTOR, which is targeted by miR-101, led to reduced tumor growth in engrafted ALCL mouse models [587]. Overexpression of miR-101 reduced cell proliferation in ALK^+^, but not in ALK^−^ [587]. The former was also more sensitive to mTOR inhibition by the rapamycin analogue CCI-779 [587]. miR-29a and miR29b down-regulation in ALK^+^ ALCL confer apoptotic resistance due to Mcl-1 upregulation [380, 587].

Another microRNA that has been implicated in NPM-ALK-driven oncogenicity is miR-135b [588]. miR-135b targets FoxO1 and promotes a IL-17-producing immunophenotype. miR-135b inhibition reduced tumor angiogenesis and growth in vivo, suggesting that targeting this microRNA has therapeutic potential [588].

#### 3.2.5. miRNAs in Diffuse Large B-Cell Lymphoma (DLBCL)

A 9-miRNA signature (miR-146b-5p, miR-146a, miR-21, miR-155, miR-500, miR-222, miR-363, miR-574-3p, and miR-574-5p) could differentiate the diffuse large B-cell lymphoma (DLBCL), the most common subtype of non-Hodgkin's lymphoma, into ABC (activated B-cell) or GCB (germinal center B-cell) subtypes, with a general higher expression in the ABC subtype [533]. Another study [780] found that miR-331, miR-151, miR-28, and miR-454 were upregulated in the GCB type, whereas miR-222, miR-144, miR-451, and miR-221 upregulated in the ABC type. The microRNA expression of both GCB-like and ABC-like cells was more similar to germinal center lymphocytes than memory B-cells [533]. The region encoding the miR-17~92 cluster was more commonly amplified in GCB-like than ABC-like DLBCL [781]. Lawrie et al. [580] identified 3 miRNAs, miR-155, miR-21, and miR-221, more highly expressed in ABC type than GCB type cells. Expression of miR-21 was an independent prognostic indicator in DLBCL [580]. Expression of miR-155 and miR-21 was also higher in nonmalignant ABC than in GCB cells [580]. miR-150 was strongly downregulated in both ABC and GCB-like DLBCL cells [533]. Patients with GCB DLBCL have longer overall survival and event-free survival compared with patients with an ABC phenotype when treated with R-CHOP [782, 783]. Increased expression of miR-18a in DLBCL was associated with a shorter OS (overall survival) of patients receiving R-CHOP regimen [693]. Increased expression of miR-181a was associated with longer PFS (progression-free survival), while increased expression of miR-222 was associated with shorter PFS [693]. In DLBCL, miR-181a regulates FoxP1 (Forkhead Box protein P1) and MGMT (O^6^-methylguanine-DNA methyltransferase) expression in DLBCL cells [693]. FoxP1 is expressed in normal activated B cells, mantle zone B cells, and some germinal center B cells [784, 785]. FoxP1 is recurrently targeted by chromosomal translocations involving the immunoglobulin heavy chain locus in marginal zone lymphomas and DLBCL, suggesting a potential role for FoxP1 in lymphomagenesis [786, 787]. FoxP1 has in some studies been shown to be associated with poor prognosis and survival [788, 789]. MGMT encodes an enzyme that protects cells from the toxicity of alkylating agents. The ability of miR-181a to reduce MGMT protein expression may contribute to better cyclophosphamide chemosensitivity [693].

miR-222 is part of the miR-221/miR-222 cluster, which is highly expressed in ABC-like DLBCL cell lines [533] and ABC-like DLBCL tumors [580]. miR-222 regulates the expression of the stem cell factor c-Kit [697], and the cyclin-dependent kinase inhibitors p27^Kip1^ and p57^Kip2^ [695, 698]. High expression of miR-222 was associated with inferior overall survival and progression-free survival [533].

#### 3.2.6. MicroRNA in Follicular Lymphoma (FL)

FL is characterized by high miR-9, miR-138, miR-20a/b, and miR-155 expression [135, 564, 582]. 

*3.2.6.1. miR-9 in FL*. miR-9, which is activated by c-Myc, regulates NF*κ*B [641]. miR-9 targets also the transcription factor PRDM1/Blimp1 in lymphoma and may contribute to the phenotype maintenance and pathogenesis of lymphoma cells by interfering with normal B-cell terminal differentiation [582, 608]. BRDM1/Blimp1 has been considered to be a tumor suppressor [790, 791]. Besides miR-9, let7a and miR-125b regulate BRDM1/Blimp1 expression [533, 640]. BRDM1/Blimp1 and Bcl6 are critical regulators of germinal center B-cell differentiation [594, 792, 793]. BRDM1/Blimp1 and Bcl6 are expressed in a mutual exclusive pattern and evidence suggests that they repress each other in germinal center B cells [792, 794]. A marked decrease of BRDM1/Blimp1 and an increase of Bcl6 were observed in follicular lymphoma cells [135], which might be related to the increased miR-9 levels in these cells [564]. Mutations in BRDM1/Blimp1 are frequently found in activated B cell (ABC)-like DLBCL [790, 795].

#### 3.2.7. miRNAs in Hodgkin's Lymphoma (HL)

The malignant Hodgkin's lymphoma cells are usually derived from B cells, but have lost the expression of typical B-cell genes. Multiple signaling pathways are deregulated, including NF*κ*B, JAK (Janus kinase)/STAT (signal transducer and activator of transcription), PI3K/Akt, ERK, Notch1, and receptor tyrosine kinases [796]. Patients with low miR-135a expression had a higher probability of relapse and a shorter disease-free survival [677]. miR-135a targets JAK2, a cytoplasmic tyrosine kinase involved in a subset of cytokine receptor signaling pathways. Transfection of pre-miR-135a into classical HL (cHL) caused apoptosis and decreased cell growth [677]. The miR-135a-mediated JAK2 down-regulation led to decreased Bcl-X_L_ expression [677], a downstream effector of JAK2 [797].

About 40%–60% of Hodgkin's lymphomas have EBV (Epstein-Barr virus) associated with the malignant cells. EBV could transactive miR-155 through NF*κ*B activation [689]. Since miR-155 is overexpressed in Hodgkin's lymphoma [590] and promotes B-cell lymphoma formation [602, 798, 799], EBV may be important in the pathogenesis of cHL.

## 4. MicroRNA in Regulating GC-Induced ****Apoptosis of Lymphoid Malignancies

### 4.1. MicroRNAs in the Regulation of GR Expression

#### 4.1.1. Downregulation of GR by miR-18 and miR-124a

MicroRNAs have been shown to modulate GR expression in neuronal tissue [649, 800, 801]. miR-18 and miR-124a especially reduced GR-mediated events in addition to decreasing GR protein levels [649]. miR-18 is part of the miR-17~92 cluster, which is repressed by GCs [206]. Upregulation of the miR-17~92 has causally been related to small cell lung cancer [802, 803], where reduced GR levels have been associated with GC resistance [804].

miR-124a was found to bind to the 3′ untranslated region of GR mRNA [649]. Activation of the GR-responsive glucocorticoid-induced leucine zipper (GILZ) was impaired by miR-124a and -18 overexpression, while miRs-22, -328, and -524 did not have any effect [649]. Of note, miR-124 regulates *Hes1* expression in P19 teratocarcinoma cells [670], a transcription factor that negatively regulate GR expression [88]. GC resistance in sepsis patients was associated with miR-124-induced downregulation of GR [805].

#### 4.1.2. Downregulation of GR by miR-130b

While miR-130b, -181a, and -636 have putative complimentary binding sites in the 3′-UTR of GR*α*, only miR-130b was found to down-regulate endogeneous GR protein expression in the multiple myeloma cell line MM.1 [674]. The miR-130b, -181a, and -636 were differentially expressed between GC-sensitive and GC-resistant MM.1 cell lines [674]. miR-130b was expressed at higher levels in the resistant MM cell line [674]. Overexpression of miR-130b in MM.1S cells resulted in decreased expression of endogeneous GR, decreased induction of the GR-target gene GILZ, and induction of GC resistance [674]. Expression of miR-130b was therefore suggested to be a potential biomarker for patients who could be refractory to GC therapy.

In gastric cancers, miR-130b regulated the tumor suppressor gene RUNX3 [675]. miR-130b may also down-regulate p21^Waf1/Cip1^, resulting in inhibition of cellular senescence [676, 806].

#### 4.1.3. Downregulation of GR by miR-142 and miR-181a

Another study [678] showed that elevated miR-142 expression in human T-ALL cells confers GC resistance by reducing the GR expression level. Other mechanism for the oncogenic role of miR-142 might be explained by its targeting of adenylyl cyclase 9 mRNA [679] leading to reduced production of cyclic adenosine monophosphate (cAMP) production with concomitant inhibition of the protein kinase A (PKA) signaling pathway [678]. The reduction in cAMP levels and reduced PKA activity caused by miR-142 relieve the inhibitory effect of PKA on T-leukemic cell proliferation. T-ALL with poor prognosis expressed higher levels of miR-142 than those with good prognosis [678]. Also, miR-142 was expressed at higher levels in relapsed T-ALL than newly diagnosed samples [678]. Transfection of miR-142 inhibitor increased GR*α* expression levels and sensitized T-ALL cells to GC-induced apoptosis [678].

These findings are in accord with previous findings showing a synergistic effect of cAMP mimetics on GC-induced apoptosis [99, 460, 807]. cAMP signaling can also be negatively regulated by phosphodiesterase 4B (PDE4B) that is frequently overexpressed in diffuse large B-cell lymphoma (DLBCL) [808]. Pharmacological inhibition of PDE4 in a xenograft model of human lymphoma unleashed cAMP effects, inhibited Akt, and restored GC sensitivity [808]. PDE4 inhibitors may thus improve the clinical outcome of patients with B-cell malignancies.

Triptolide, a drug that overcomes dexamethasone-resistance in human multiple myeloma cells [809], was found to regulate GR expression in the MM1.S cell line by downregulating the expression of miR-142 and miR-181a [680]. miR-142 and miR-181a mimetics slightly attenuated, whereas miR-142 and miR-181a inhibitors enforced GC-induced apoptosis of MM1.S cells [680]. miR-181a/b can also increase GC-induced apoptosis in virtue of their ability to repress the expression of the anti-apoptotic Bcl-2, Mcl-1, and XIAP proteins [385, 539, 810]. 

### 4.2. MicroRNAs Affected by GCs in Lymphoid Cells

#### 4.2.1. Repression of miR-17~92 by GCs

Smith et al. [648] showed that broad microRNA repression occurs during GC-induced apoptosis of rat thymocytes. This repression was associated with reduced expression of both nuclear (Drosha and DGCR8/Pasha) and cytoplasmic (Dicer) microRNA processing enzymes. Silencing of Dicer in two human leukemic cell lines (CEM-C7 and ectopic GR*α*-overexpressed Jurkat cells) led to enhanced sensitivity to GC-induced apoptosis [648]. Global downregulation of microRNA levels, especially the miR-17 family, by GCs was also observed in GC-sensitive ALL cell lines, with concomitant upregulation of Bim [657]. Later studies showed that GCs selectively upregulate and downmodulate specific miRNAs [646] that cannot be explained by altered Dicer expression.

One polycistron cluster repressed by GCs is miR-17~92 [648, 657], which regulates Bim expression [246, 247]. Downregulation of miR-17~92 contributes to the GC-mediated upregulation of Bim [206]. This microRNA cluster also represses PTEN [247], a negative regulator of the PI3K/Akt signaling pathway. The GC-mediated downregulation of miR-17~92 might be one mechanism responsible for the GC-induced dephosphorylation of Akt. Primary thymocytes derived from mice transgenic for the miR-17~92 polycistron members in the lymphocyte compartment exhibited diminished sensitivity to GC-induced apoptosis in lymphocytes, further supporting a role for GC-induced repression of miR-17~92 in promoting apoptosis [648]. Harada et al. [657] observed that GCs reduced miR-17 family expression in 50% of primary GC-sensitive ALL, but not in any of the GC-resistant ones. Overexpression of miR-17~92 attenuated GC-induced cell death, while inhibition of miR-17~92 increased the sensitivity to GC [657]. They also reported that in a pre-B ALL cell line, a 10-hour dexamethasone treatment led to a reduction in miR-142 and miR-27a, while miR-9 was induced. There is also some evidence that GCs can reduce miR-27a expression in mouse muscle cells [811].

#### 4.2.2. Upregulation of miR-15~16 by GCs

Rainer et al. [646] reported an induction of the myeloid-specific miR-223 and the apoptosis and cell-cycle arrest inducing miR-15~16 clusters by GC in a subset of B- and T-ALL cells, together with downregulation of the miR-17~92 complex. A transient upregulation of miR-19b and miR-181a was also observed. Overexpression of miR-15b~16 mimics increased, whereas silencing by miR-15b~16 inhibitors decreased GC sensitivity [646]. The miRNAs of the miR-15~16 family are encoded in two clusters (15a~16-1 and 15b~16-2) embedded in the DLEU2 (deleted in leukemia 2) and SMC4 loci, respectively [766, 812]. They have been implicated in cell-cycle arrest [813] and in cell death/survival decisions, the latter supposedly by targeting Bcl-2 [346]. Other microRNAs affected by GCs in pediatric ALL include upregulation of miR-548d-1 and repression of miR-128b along with miR-106b~25~93, the paralogue of miR-17~92 [646].

#### 4.2.3. Upregulation of miR-223 by GCs

It is still not known whether the GC-induced upregulation of miR-223 affects GC-induced apoptosis [646]. Increased expression of miR-223 is involved in the differentiation of myeloid precursors into granulocytes such as neutrophils [701, 814]. During granulopoiesis, miR-223 targets E2F1, which in turn represses miR-223 expression, creating an autoregulatory negative feedback loop [702]. A negative feedback loop also exists between miR-223 and the transcription factor NFI-A [814]. miR-223 is positively regulated by C/EBP*α* during differentiation to granulocytes [814] and negatively regulated by AML1/ETO in leukemia cells [703]. Moreover, miR-223 targets the myeloid ELF-1-like factor (Mef)-2c and IGFR (insulin-like growth factor receptor), which may account for some of its negative regulation of granulocyte proliferation [701]. Through suppression of IGF-1R, the downstream PI3K/Akt/mTOR/p70S6K pathway is suppressed, with consequent inhibition of cell proliferation [704]. miR-223 attenuates hematopoietic cell proliferation and positively regulates miR-142 through LMO2 isoforms and C/EBP*β* [815]. Ectopic expression of miR-223 restores differentiation of AML leukemic cells [703]. miR-223 knockout mice showed increased numbers of granulocyte progenitors in the bone marrow and hypermature neutrophils in the circulation, suggesting that miR-223 is involved in the negative regulation of maturation rather than differentiation of granulocytes [701]. miR-223 may also target Fbw7 [705, 816], a negative regulator of the anti-apoptotic Mcl-1 [372]. Thus, it may indirectly increase apoptotic resistance by up-regulating Mcl-1.

#### 4.2.4. Upregulation of miR-150 and miR-342 by GCs

Dexamethasone treatment of thymocytes led to upregulation of miR-150 and miR-342, while miR-181a and miR-181d were downregulated [684]. miR-181d represses CD69 and Prox-1 to a similar extent as miR-181a [684]. miR-181d, but not miR-181a, repressed Lif (leukemia inhibitory factor) [684]. Lif is a member of the IL-6 cytokine family expressed in thymic epithelial cells and T lymphocytes, which elevates GC levels following LPS exposure and is responsible for thymic atropy induced by stress [817–819]. Other effects of miR-181 are described in Sections 3.1.3 and 3.2.2.2. The effects of miR-150 are described in Sections 3.1.4 and 3.2.2.9.

#### 4.2.5. Effect of GCs on MicroRNA Expression in Macrophages

A recent report showed that GCs could prevent lipopolysaccharide (LPS)-mediated inflammatory responses in macrophages by downregulating miR-155 [690]. LPS induces miR-155 expression in macrophages through TLR4-mediated activation of NF*κ*B [690]. Overexpression of miR-155 reversed the suppressive action of GCs, while inhibition of miR-155 exhibited an effect similar to that of GCs on LPS-treated macrophages, suggesting that GC-induced repression of miR-155 is one mechanism for the immunosuppressive function of GC. This effect of GC on miR-155 was dependent on GR and NF*κ*B [690]. miR-155 transgenic mice produced more proinflammatory cytokines in response to LPS [820]. miR-155 is transcribed from B-cell integration cluster (BIC) [584, 691] and targets among others SOCS1 (suppressor of cytokine signaling 1), which negatively regulates JAK/STAT signaling. GCs also prevented the LPS-mediated upregulation of miR-146, miR-147, miR-148, miR-32b, and miR-301 in macrophages [690].

#### 4.2.6. Other MicroRNAs Affected by GCs

In the brain, GCs prevents BDNF (brain-derived neurotrophic factor)-regulated synaptic function through suppression of miR-132 expression [821]. miR-132 is increased by BDNF and is involved in promotion of neuronal outgrowth [822]. In some carcinoma cell lines, dexamethasone was shown to down-regulate miR-27b, miR-148a, and miR-451 [823].

### 4.3. MicroRNAs in the Regulation of Apoptotic GC-Sensitivity

**Figure 6:**
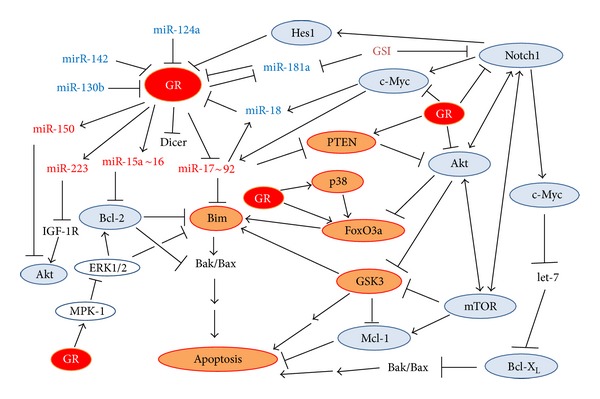
The complexity of GC-induced apoptosis. A summary of the main issue discussed in this paper.

From all we have learned above, any microRNA that modulates any of the many factors regulating GC-induced apoptosis may affect the apoptotic response to GCs (Figures 1–6). These include microRNAs that affect GR expression (e.g., miR-18, miR-124a, miR-130b, miR-142, and miR-181a), those affecting Bim expression (miR-26a, miR-93, miR-17~92, miR-106a~363, and miR-106b~25) or its transcription factor FoxO3 (e.g., miR-1, miR-21, miR-27a, miR-96, miR-135b, miR-155, and miR-182), those affecting PTEN expression (miR-17~92, miR-106b~25, miR-21, miR-26a, miR-29b, miR-214, miR-216a, miR-217, miR-221, and miR-222) or mTOR (e.g., miR-101), and those downregulating directly or indirectly the anti-apoptotic proteins Bcl-2, Bcl-X_L_, Mcl-1, XIAP, and CYLD (e.g., miR-15a~16, miR-181a/b, miR-34a, miR-125b, miR-29a/b/c, miR-101, miR-133b, miR-193b, miR-512, let-7, and miR-491). The effect of some of these microRNAs on GC-sensitivity has already been described above and will not be repeated here. Rather, I will present here some data from primary samples showing the influence of microRNAs on clinical outcome.

A study searching for differential miRNAs expression in ALL relapse cells versus childhood ALL with complete remission showed significant associations for miR-708, miR-223, and miR-27a with individual relapse-free survival [655]. For samples at relapse versus diagnosis, the most differentially expressed microRNAs included miR-223, miR-23a, let-7g, miR-181, miR-708, and miR-130b, while comparison of complete response with diagnostic samples showed differential expression pattern of miR-27a, miR-223, miR-23a, miR-181, and miR-128b [655]. Among these microRNAs, miR-223, miR-128b, miR-23a, and let-7g were downregulated in the relapse samples compared with complete response samples, while miR-181 family members, miR-708, and miR-130b were upregulated in the relapse samples [655]. It should be remained here that miR-130b targets GR [674], RUNX3 [675], and p21 [676], and miR-223 is upregulated by GCs [646] and targets IGFR [701] and E2F1 [702]. E2F1 has a dual role in cell-cycle control, as it affects several cell processes. It can either act as a tumor-suppressor or oncogene depending on the cellular context [824]. Thus, the upregulation of miR-130b together with downregulation of miR-223 may contributes to GC resistance.

miR-708 was the most upregulated microRNA in the relapse samples, whereas miR-223 was significantly downregulated, suggesting that these two microRNAs may have important role in pediatric ALL relapse [655]. Moreover, upregulation of miR-708 was found to be associated with the in vivo GC therapy response and with disease risk stratification in childhood ALL [655]. Standard and middle risk stratification groups had a higher miR-708 expression at diagnosis than the high risk group. Interestingly, miR-708 was low in high relapse patients at diagnosis, while specimens of relapsed samples showed abundance of miR-708, suggesting for an upregulation of miR-708 during disease progression.

FoxO3, that is critical for hematopoietic stem cell self-renewal and mediates the initial apoptotic response [825–827], contains a conserved miR-708 response element in its 3′-UTR [655]. FoxO3 can act as either an oncogene or a tumor suppressor in leukemia [828, 829]. FoxO3 transcriptional activity was found to prevent B-CLL and CML proliferation [825, 828]. FoxO3a is also targeted by other microRNAs, including miR-27a (see Section 2.2.6).

Moreover, miR-27a directly regulates the drug-resistant factor P-glycoprotein, and overexpression of miR-27a increased sensitivity of leukemia cells to doxorubicin [658]. miR-27a is relevant to treatment outcome in vivo and may be involved in relapse of both lymphocytic leukemia and myeloid leukemia [658]. Low expression of miR-27a might promote ALL relapse [655, 658]. On the contrary, miR-27a exerts oncogenic effects by regulating ZBTB10 [446, 830] and Fbw7 [253, 438].

miR-128b, which was higher in relapse ALL and at diagnosis compared to complete response [655], has been reported to confer drug resistance in many cancers including ALL [672, 673]. Both miR-27a and miR-128b might target BMI1 [655], a transcription factor of the polycomb-group gene necessary for hematopoietic stem cell (HSC) and leukemia stem-cell self-renewal [831, 832]. Deletion of BMI1 inhibits self-renewal of tumor stem cells and prevents leukemia recurrence [833].

A role for miR-128 and miR-221 in regulating GC sensitivity in cells from MLL-AF4 ALL patients has been proposed [672]. miR-128b and miR-221 are downregulated in MLL-arranged ALL relative to other types of ALL [672]. The *MLL* gene is located at 11q23, a site frequently involved in chromosomal translocations in aggressive human lymphoid and myeloid leukemias. As a result of chromosomal translocations, a portion of MLL becomes fused to one among more than 40 different partner proteins. MLL-AF4 ALL, which results from the translocation between MLL and AF4, is associated with GC resistance and has a poor prognosis [834, 835]. Re-expression of mR-128 and miR-221 in cultured MLL-AF4 ALL cells sensitized them to GCs [672]. miR-128 targets MLL, AF4, and the MLL-AF4 pusion protein resulting in lower expression of HOXA9, whereas miR-221 downregulates *CDKN1B* (cyclin-dependent kinase inhibitor 1B, p27^Kip1^), another gene transcriptionally activated by MLL-AF4 as well as the wild-type MLL protein [672]. The targeting of different proteins may explain the cooperative effect of miR-128b and miR-221 on GC sensitization [672]. It should be noted that miR-221 in other settings, for example, CLL, has anti-apoptotic effects and functions as an oncogene.

### 4.4. Potential Use of miRNA Regulators in Therapy of Cancer Cells

In light of the multiple effects of various microRNAs on cell survival and apoptosis, modulating microRNA expression in tumor cells is an attractive approach for sensitizing the tumor cells to chemotherapeutic drugs. Inhibition of specific microRNAs is performed by using antisense sequences (termed antagomiRs) targeting the microRNA guide stand that blocks the interaction with the microRNA recognition elements within the 3′-UTR of the target mRNA genes [836]. To increase their binding affinity and stability in biological fluids, the antagomiRs are often modified with 2′-O-methyl-, phosphorothioate, or locked nucleic acid substitutions. To overexpress microRNAs, chemically synthesized microRNAs (called microRNA mimics) are used.

One potential use of microRNAs is to repress the expression of MLL-AF4 fusion protein in ALL that is responsible for GC resistance. This fusion protein can be repressed through overexpression of miR-143 [837], or miR-128 together with miR-221 [672]. The latter combination was shown to sensitize the MLL-AF4-carrying ALL cells to GCs [672]. 

Another promising approach is to target miR-155, an oncogenic microRNA often correlated with poor prognosis. A proof-of-principle was demonstrated by Babar et al. [838]. They showed that overexpression of miR-155 in lymphoid tissues resulted in disseminatedlymphomacharacterized by a clonal, transplantable pre-B-cell population of neoplastic lymphocytes. Withdrawal of miR-155 in mice with established disease resulted in rapid regression of lymphadenopathy. Systemic delivery of antisense peptide nucleic acids encapsulated in unique polymer nanoparticles inhibited miR-155 and slowed the growth of these pre-B-cell tumors in vivo [838].

## 5. Summary

Glucocorticoid-induced apoptosis appears to be a complex process involving several signaling pathways (Figure 6). These include (1) transactivation of pro-apoptotic genes (importantly Bim); (2) alterations in microRNA expression (upregulation of miR-15~16 that targets the pro-apoptotic Bcl-2; miR-223 that targets IGFR; miR-150 that targets Akt and Notch, while suppressing miR-17~92 that prevents Bim and PTEN translation); (3) direct action of GR on the mitochondria (including mitochondrial GR translocation and production of reactive oxygen species within the mitochondria); (4) activation of the protein kinases GSK3 and p38; (5) activation of the FoxO3a transcription factor that upregulates Bim; (6) inhibition of the Notch1, PI3/Akt/mTOR, and ERK1/2 survival pathways. Interruption of any of the pro-apoptotic processes may lead to drug resistance. Altered microRNA expression in malignant cells may modulate many of these processes thereby imposing apoptotic resistance (Figures 1–6).

GC-resistant lymphoid cells might be divided into two major subgroups according to the underlying mechanism of resistance. The first group consists of cancer cells whose drug resistance can be overcome by exposing the cells to GCs in combination with drugs that target protein kinases such as Akt, mTOR, Src, ALK, and/or BCR, or drugs antagonizing Bcl-2, Bcl-X_L_, Mcl-1, c-Myc, or Notch. These lymphoid malignancies show in general a more favorable response to combined GC therapy and in many cases may be explained by their growth dependency on these signaling molecules. The second group of GC-resistant cells exhibits an intrinsic defect in the GC-mediated apoptotic process and can thus not be turned sensitive to this drug. It is important to distinguish between these two subgroups prior to therapy initiation in order to choose the right drug combination. A diagnostic test needs to be developed that can distinguish between the different resistance backgrounds.

Recently, Burnsides et al. [839] have developed an ex vivo stimulation assay that determines the ability of leukocytes to upregulate anti-inflammatory genes such as GILZ and FKBP51 following exposure to dexamethasone. It is reasonable that a similar test may be developed to gene profiling lymphoid malignancies prior to and following GC treatment, where upregulation of the pro-apoptotic *Bim* gene would be a favorable predictor. Also, Bim induction may be measured after combining GC with a protein kinase inhibitor. Simultaneous expression profiling of microRNAs, Notch1, and Bcl-2 family proteins together with the activated protein kinase status in the malignant cell would provide valuable information for choosing the proper drug combination. A predictor for a good GC response would be to determine the ability of GCs to downregulate miR-17~92 and upregulate miR-15~16, miR-150, and miR-223.

A tentative therapeutic approach would be to modulate the microRNA status of the cell using microRNA mimics or antagomiRs as described in Section 4.4. What we have learned from the studies described in this paper is that it seems that in general it would be favorable to augment the expression of miR-29, miR-27, miR-15a~16, miR-34a, miR-150, and let-7, while suppressing miR-155, miR-181, miR-182, miR-21, and miR-221/222 as well as miR-17~92. Obviously, an initial microRNA profiling should be performed, and the cancer-type classification should be considered. Some microRNAs may have cell-type specific effects. While down-regulation of miR-181 may suppress the growth of T-ALL and MM, augmented miR-181 expression prevents the growth of unmutated IgVH CLL cases. Also, miR-26a has a dual effect. Its overexpression prevents growth of c-Myc-positive Burkitt lymphoma, while it must be downregulated in Notch-positive T-ALL to achieve growth inhibition. miR-451 and miR-709 could prevent growth of Notch-positive T-ALL. A reduction in miR-142, and maybe also of miR-708, which is highly expressed in relapsed childhood T-ALL, is anticipated to improve T-ALL therapy. For classical HL, miR-135a may cause apoptosis.

In conclusion, in certain types of lymphoid malignancies, GC resistance may be overcome by relieving the inhibitory effects of protein kinases and Bcl-2 family members. Both the activity of protein kinases and the expression of Bcl-2 members are affected by the microRNA network. Modulation of microRNA expression might increase GC drug responsiveness and thus improve the therapy of lymphoid malignancies.

## Figures and Tables

**Table 1:** Alterations in microRNA signature during T-cell development in the thymus. (according to Neilson et al. [539]).

Thymocyte population	Relative high expression	Relative low expression
DN1	miR-21, miR-29b, miR-342, miR-221, miR-223	miR-16, miR-128b, miR-15b, miR-24
DN3	miR-191	miR-142, miR-150
DN4	miR-142, miR-20a, miR-16, miR-128b	
DP	miR-92, miR-181a/b, miR-350, miR-15b, miR-16	miR-21, miR-27a
CD4^+^ SP	miR-669c, miR-297, miR-142	miR-142
CD8^+^ SP	miR-15b, miR-150, miR-24, miR-27a, miR-142	miR-142, miR-16, miR-128b, miR-92, miR-181b

DN: double negative (CD4^−^8^−^)

DP: double positive (CD4^+^8^+^)

SP: single positive (CD4^+^8^−^ or CD4^−^8^+^).

**Table 2:** MicroRNA signature in various lymphoid malignancies. The table shows microRNAs that have been detected at higher or lower levels in lymphoid malignancies according to data in the literature. More detailed description is found in Sections 3 and 4. It should be emphasized that the table presents microRNAs that are frequently dysregulated, and the microRNA expression pattern may vary during disease progression and depends on the ontogeny and tumor grade. Also, there are variabilities between the different studies which may be due to generalized classification or more specific classification of the given malignancy. Also, the reference gene and cell type used as control may affect the interpretation of microRNA profiling. MicroRNAs that can affect or are related to GC signaling and/or GC-induced apoptosis are highlighted in bold.

Cancer type	Increased expression	Decreased expression	References
ALL	**miR-17**~**92 cluster**, **miR-26a**, miR-29a/b/c, miR-125b-1*, miR-128a, miR-128b, **miR-146a**, miR-204, miR-218, miR-331, **miR-181a**, **miR-181b**, miR-181c, miR-142-3P, **miR-142**, **miR-150**, **miR-155**, miR-193a, miR-196b, miR-30e-5p, miR-34b, miR-365, miR-582, miR-708, miR-223*	**let-7b**, miR-223*, miR-100, miR-125b*, miR-151, miR-99a, miR-124a	[253, 557–562]

CLL	**miR-21**, miR-23b, miR-24-1, miR-146a, miR-150*, **miR-155**, miR-106b, miR-195, miR-221*, miR-222*, **miR-181a**/b*, miR-19a, miR-20a, miR-106b, miR-142*, miR-29a/c*, miR-130, miR-26a, miR-197, miR-342, miR-483, miR-595	**miR-15a, miR-16-1**, miR-29*, **miR-34a**, miR-143, miR-45, miR-30d, **let-7a**, miR-181a/b*, **miR-223**, miR-92, miR-150*, miR-126, miR-125b, miR-103, miR-572, miR-494, miR-923, miR-130a, miR-213, miR-17, miR-142*, miR-206, miR-220, miR-221*, miR-222*, miR-182, miR-199a, let7, miR-424, miR-10a, miR-7, miR-126, miR-218	[345, 346, 358, 557, 558, 563–573]

MM	**miR-21**, **miR-106b**~**25 cluster**, miR-181a/b*, miR-20a, miR-19a, miR-19b, miR-93, miR-25, miR-92a, **miR-19a, miR-19b**, miR-32, miR-1, miR-133a, miR-193b~365,	**let-7b**, let-7-1, let-7c, miR-29a, and miR-29b, miR-328, miR-15a/16, miR-192~194~215, miR-181a/b*	[345, 574–579]

DLBCL	**miR-155**, miR-124a miR-125b*, miR-143, miR-451, miR-145, miR-10b, miR-34a, miR-100, miR-9, miR-21, miR-17~92, miR-128a, miR-106a/b, miR-425, **miR-130b**, miR-181b*	miR-27a/b, miR-29a/b/c; miR-142, **miR-150**, miR-125b*, miR-101, miR-28, miR-16, miR-189, miR-363, miR-223, miR-584, miR-361, miR-768, miR-625, miR-495, miR-181a*, miR-189, miR-363, miR-595, miR-663	[533, 564, 580–585]

C-ALCL	**miR-155**, miR-27b, miR-30c, miR-29b		[586]

ALK^+^-ALCL	miR-886-3p, miR-17, miR-18a, miR-20a, miR-363, miR-106a, miR-20a, miR-20b, miR-135b	miR-146a, miR-101, miR-29b, miR-26a, miR-29c, miR-29a, miR-22, miR-150, miR-125b	[587, 588]

ALK^−^-ALCL	miR-155	miR-101	[587]

cHL	**miR-17**~**92 cluster**, miR-16, miR-21, miR-24, **miR27a**, miR-124a, miR-134, miR-138, **miR-155**, miR-147, miR-182, miR-185, miR-198, miR-216, miR-220, miR-302a/b/c, miR-325	miR-23b, miR-30b, miR-31, miR-96, miR-126, miR-128a/b, **miR-135a**, miR-183, miR-204, miR-205, miR-335, **miR-150**	[584, 589, 590]

MCL	**miR-124a**, **miR-155**, **miR-182**, miR-183, miR-328, miR-326, miR-302c, miR-345, miR-373, miR-210, miR-617, miR-370, miR-654, miR-106b, miR-93, miR-25, miR-200c, miR-363, **miR-181c**, miR-654, miR-768	miR-29a/b/c, miR-142, **miR-150**, **miR-15a/b**, miR-31, miR-148a, miR-27b, miR-126	[564, 583, 591]

FL	miR-9, **miR-20a/b**, miR-301, miR-213, miR-330, miR-106a, miR-338, **miR-155**, miR-210, miR-138, miR-193a, miR-345, miR-513b, miR-574, miR-584, miR-663, miR-1287, miR-1295, miR-1471	miR-30a, miR-33a, miR-106a, miR-141, miR-202, miR-205, miR-222, miR-301b, miR-320, miR-149, miR-139, miR-431, miR-570	[135, 564, 582]

Abbreviations: ALK: anaplastic lymphoma kinase; ALCL: anaplastic large cell lymphoma; C-ALCL: cutaneous large cell lymphoma; cHL: classical Hodgkin's lymphoma; DLBCL: diffuse large B-cell lymphoma; FL: follicular lymphoma; MCL: mantle cell lymphoma.

*Variation in expression, dependent on the tumor grade.

**Table 3:** Target genes of prominent microRNAs in lymphoid malignancies and their role in regulating GC-mediated apoptosis. Relations to GC signaling and/or GC-induced apoptosis are highlighten in bold. More detailed description is found in Sections 3 and 4.

miRNA	Important target genes	Regulation/expression	Effect on GC-induced apoptosis	References
let-7 family	K-Ras, Myc, HMGA2, PLC*γ*1, IMP-1, Dicer, IL-6, E2F2, CCND (Cyclin D2, Cdc25A, CDK6, **Bcl-** **X**_**L**_, PRDM1/Blimp1	↓ CLL ↓ MM ↓ T-ALL ↓ BL ↓**Myc**	Anticipated to synergize with GC	[345, 361, 473, 476–479, 525, 564, 640]
miR-9	PRDM1/Blimp1 NF*κ*B	↑ FL		[564, 640–642]
miR-15a~16	**Bcl-2**, CHEK1, CCND1 (Cyclin D1), CCND2 (Cyclin D2), CCND3 (Cyclin D3), CCNE (Cyclin E), CDK4, CDK6, Wnt3a, E2F, Cdc25A, Mcm5	↑↓ CLL ↓ MM ↑**GC** ↑**E2F1-3** ↓**c-Myc**	Promote GC-induced apoptosis	[255, 345, 346, 471, 473, 515, 525, 564, 566, 576, 643–646]
miR-17~92 cluster	**Bim (Bcl2L11),** **PTEN**, E2F1, **Notch1**, Hoxa9, **CYLD**, **RUNX1,** p21	↑ T-ALL ↑ CLL ↑ MM ↑ BCL ↑ ALK^+^-ALCL ↑ DLBCL ↑ BL ↓**GC** ↑ c-Myc ↑ E2F1 ↓ GSI	Attenuates GC-induced apoptosis. Considered as an OncomiR.	[135, 206, 246–248, 255, 445, 467, 469, 515, 525, 564, 574, 618, 647, 648]
miR-18 (member of the miR-17~92 cluster)	**GR**		Reduced GR-mediated transactivation	[649]
miR-21	**PTEN**, PDCD4, TPM-1, Tap63, SPRY2, Msh2, SHIP1, TRAIL-3	↑ CLL ↑ CML ↑ MM ↑ BCL ↑ DLBCL ↓**FoxO3a**	Expected to prevent GC-induced apoptosis, due to increased Akt signaling. Considered as an OncomiR.	[268, 269, 525, 566, 574, 579, 580, 650–654]
miR-23a/b	**Notch1**, PLK3, PAX, MTSS1	↑ CLL ↓ cHL ↓ Relapsed T-ALL		[590, 655, 656]
miR-26a	**PTEN**, **Bim**, EZH2, c-Myc, CCND3 (Cyclin D3), CCNE2 (Cyclin E2)	↑ T-ALL ↑ CLL ↓ BL ↓ Myc	Expected to prevent GC-induced apoptosis. Considered as an OncomiR.	[253, 315, 465, 473, 481, 565, 566, 629]
miR-27a	Fbw7, ZBTB10, Myt-1, MDR, BMI1, **FoxO1/3**	↑ B-ALL ↓ DLBCL ↓**GSI** ↓**GC**		[253, 258, 438, 439, 445, 446, 657, 658]
miR-29a/b	**Mcl-1**, Tcl-1, CDK6, **PTEN**, DNMT1, DNMT3A, DNMT3B p85*α*, CDC42	↓ ALCL ↓ CLL ↓ MM ↓ MCL ↓ DLBCL ↓ BL ↓ Myc ↓ NF*κ*B	Expected to synergize with GC.	[316, 380–383, 473, 564–566, 569, 574, 583, 587, 643, 655, 659–662]
miR-34a/b/c	**Bcl-2**, E2F1, c-Myb, B-Myb SIRT1, ZAP70, **Notch1,** **Delta1,** **Jagged1**	↑↓ CLL ↑**p53** ↑**PMA** ↓**Myc**		[347, 348, 443, 473, 570, 663–667]
miR-101	**mTOR**, **Mcl-1**, Cox2, Fos, EZH2	↓ ALCL	Expected to synergize with GC.	[384, 587]
miR-106a~363 and miR-106b~25	p21/CDKN1a **Bim**, **PTEN**	↑ MCL ↑ MM ↑ DLBCL ↓**GC**	Attenuates GC-induced apoptosis	[254–256, 314, 574, 591, 668, 669]
miR-124a	**GR**	↑ MCL ↓ ALL	Reduced GR-mediated transactivation	[560, 583, 649, 670]
	CDK6 Hes-1			
miR125a	PDPN, **Bak1**, KLF13, preproET1, ARID3B, HuR, ERBB2, ERBB3			[576]
miR-125b	IRF4 PRDM1-Blimp1 Lin28, STAT3 **Bak1**, **Bmf** **Mcl-1**, **Bcl-w**, **Bcl-2**	↓ CLL	It has both pro- and anti-apoptotic effect.	[349–352, 356, 533, 571, 595, 671]
miR-128b	BMI1	↓ Relapsed T-ALL ↓ MLL-AF4-ALL ↓**GC**	miR-128 sensitizes MLL-AF4 ALL to GC.	[646, 655, 672, 673]
miR-130b	**GR**	↑ DLBCL ↑ Relapsed T-ALL	Attenuates GC-induced apoptosis.	[655, 674–676]
	RUNX3 p21			
miR-135a/b	JAK2,	↓ cHL		[590, 677]
miR-142	**GR**	↓ MCL ↑ T-ALL ↓**GC**	Confers GC resistance	[583, 657, 678–680]
	**AC9**			
miR-143 and miR145	MLL-ALL ERK5	↓ CLL		[525, 573]
miR-146a	TRAF6, IRAK1, Fas, Smad4, TBP, CCL8-MCP-2	↑ MM ↑ T-ALL ↑ CLL ↓ BL ↓**Myc**		[473, 564, 576, 635, 637, 638, 681]
miR-150	c-Myb, DKC1 **AKT2, Notch3**	↑↓ CLL ↑ T-ALL ↓ MCL ↓ cHL ↓ DLBCL ↑**GC** ↓ Myc		[441, 473, 547, 565, 566, 583, 589, 682–684]
miR-155	SOCS1, ETS1, c-MAF, HGAL, **FoxO3a**, SHIP1, SMAD5, PU.1, C/EBP*β*, CSFR, KPC1, CEBPB, IL-13R*α*1, CUTL1, CYR61, SMAD1, ETS1, SMAD2,	↓↑ CLL ↑ DLBCL ↑ C-ALCL ↑ ALK^−^-ALCL ↑ MCL ↑ cHL ↑ NHL	Expected to prevent GC-induced apoptosis. Considered as an OncomiR.	[263, 264, 515, 525, 564, 566, 568, 576, 583, 584, 587, 589, 599, 602, 681, 685–691]
	MEIS1, RUNX2, MYO10, PKI*α*, JARID2, AGTR1, PICALM, BACH1, ZIC3,	↑↓ BL ↓**GC** (in MΦ) ↑ NF*κ*B ↑ TLR4 ↑ c-Myb ↑ EBV		
miR-181a/b	Tcl1, Lin28, **Bcl-2, Mcl-1**, **XIAP, CYLD**, GR, CD69, TCR, Hoxa7, Hoxa9, Hoxa11, PBX3, NLK, TIMP3, Prox1, DUSP5, DUSP6, SHP-2, PTPN22, FoxP1, p27^Kip1^	↓↑ CLL ↓↑ DLBCL ↑ MM ↑ T-ALL ↓ **GC** ↓ **GSI**	Dual role on GC-induced apoptosis: attenuation through repression of GR, but sensitization due to reduced expression of anti-apoptotic proteins.	[270, 358, 359, 385, 445, 539, 546, 549, 551, 552, 565, 568, 569, 574–576, 684, 692–694]
miR-182	**FoxO1/3,** Fbw7	↑ T-ALL cell lines resistant to GC ↓ CLL ↑ MCL	Confers GC resistance.	[253, 258–261, 564]
miR-221 and miR-222	p27^Kip1^, p57^Kip2^, PTEN, TIPM3, FoxO3a, c-Kit, Puma, Dicer, APAF-1, WTAP, Ets1, Bmf, Mdm2	↑ CLL ↑ DLBCL ↑ MM ↓ MLL-AF4 ALL ↓**GSI**	Dual effect on GC-induced apoptosis. Usually oncogenic with anti-apoptotic effect. In MLL-AF4 ALL, miR-221 sensitizes the cells to GC. Considered as an OncomiR.	[321, 445, 525, 533, 563, 575, 576, 580, 672, 695–699]
miR-223	LMO2, NFI-A, MYBL1, E2F1, Fbw7, Mef2c, IGFR	↓↑ T-ALL ↓ CLL ↓ DLBCL ↓ Relapsed T-ALL ↑ **GC** ↑ C/EBP*α* ↓ NFI-A ↓ E2F1	May sensitize to GC-induced apoptosis by preventing Akt activation.	[515, 533, 565, 566, 646, 655, 700–706]
miR-708	FoxO3	↑ Relapsed T-ALL	May confer GC resistance.	[655]

↑ upregulated, ↓ downregulated.

Abbreviations: AC9: adenylyl cyclase 9; BCL: B-cell lymphoma, Blimp1: B lymphocyte-induced maturity protein 1; cHL: classical Hodgkin's lymphoma; GSI- gamma secretase inhibitor; LMO2: LIM domain only 2; MDR: multidrug resistant gene; Msh2: DNA MutS homolog 2; MTSS1: Metastasis suppressor 1; NHL: non-Hodgkin's lymphoma; NLK: nemo-like kinase; PDCD4: programmed cell death 4; PMA: phorbol myristate acetate; SHIP1: SH2 (Src-homology 2) domain-containing inositol phosphatase 1; SOCS1: suppressor of cytokine signaling; SPRY2: Sprouty2; TPM-1: Tropomyosin 1; TRAF6: TNF receptor-associated factor 6; WTAP: Wilms' tumor-associated protein isoform 1; XIAP: X-linked inhibitor of apoptosis protein.

## Abbreviations

ALCL: Anaplastic large cell lymphoma
BL: Burkitt's lymphoma
CLL: Chronic B-lymphocytic leukemia
DLBCL: Diffuse large B-cell lymphoma
FL: Follicular lymphoma
GC: Glucocorticoid
GR: Glucocorticoid receptor
GSI: *γ*-secretase inhibitor
HL: Hodgkin's lymphoma
MM: Multiple myeloma
NHL: Non-Hodgkin's lymphoma
NR3C1: Nuclear receptor subfamily 3, group C, member 1
NPM-ALK: Nucleophosmin-anaplastic lymphoma kinase
T-ALL: T-cell acute lymphoblastic leukemia.
